# Network analysis of meaning in life, perceived social support, and depressive symptoms among vocational undergraduate students

**DOI:** 10.3389/fpsyt.2025.1510255

**Published:** 2025-02-04

**Authors:** Sen sen Zhang, Wen hua Zhang, Shao hong Yong, Jia tai Chen

**Affiliations:** ^1^ Faculty of Business Administration, Guangzhou Institute of Science and Technology, Guangzhou, China; ^2^ Department of Psychology, Institute of Teacher Education, Ningxia University, Yinchuan, China; ^3^ Department of Mental Health Education, Zhoukou Vocational and Technical College, Zhoukou, China; ^4^ Business School, University of Exeter, Exeter, United Kingdom

**Keywords:** meaning in life, perceived social support, depressive symptoms, network analysis, prevention

## Abstract

**Background:**

Depression poses a considerable personal and public health problem, particularly in the post-epidemic era. The present study aimed to investigate the association between meaning in life (MIL) and perceived social support (PSS) with depressive symptoms among vocational undergraduate students, employing a network analysis approach to gain a deeper understanding of the underlying pathways and to prevent the progression of depressive symptoms into disorders.

**Methods:**

A total of 1367 Chinese vocational undergraduates (*M*_age_ = 20.1, *SD* = 1.6; 44.7% female) were recruited and were asked to complete a series of questionnaires, including the meaning in life questionnaire, perceived social support scale, and patient health questionnaire. The regularized partial correlation network was estimated. The partial correlations between nodes were calculated as edges. Moreover, network comparison tests were conducted to compare three subnetworks based on different levels of depression (minimal, subthreshold, and moderate/severe).

**Results:**

The top strength nodes within each network were identified as *sleep* and *motor* in minimal group, *anhedonia* and *concentration* in subthreshold group, and *anhedonia* and *sleep* in moderate/severe group. Additionally, the bridge strength nodes were determined as *MIL-3*, *MIL-4*, *sleep*, *guilt*, and *school* in minimal group; *MIL-4*, *anhedonia*, *suicide*, and *friend* in subthreshold group; MIL-9, *MIL-7*, *anhedonia*, *sleep*, and *family* in moderate/severe group. Furthermore, network comparison tests showed significant differences in centrality (all *p* < 0.05), while network invariance remained constant across groups. Notably, the accuracy and stability coefficients for all network structures were greater than 0.5, indicating stable and reliable results.

**Conclusion:**

These findings elucidate specific pathways and potential central nodes for interactions of MIL or PSS with depressive symptoms at different levels of depression, providing valuable insights for targeted prevention and intervention strategies.

## 1 Introduction

Depression is a significant individual and public health concern characterized by biological and psychological changes in response to stressful events (1), emerging commonly during adolescence (2). Before the COVID-19 pandemic, the prevalence of depression was reported to be 4.4% (3). However, during the pandemic, it was revealed 33.7% according to a meta-analysis of the general population’s mental health (4). The incidence of depression is rapidly increasing, notably in college student communities (5, 6). Researchers have proposed that the pandemic poses substantial threats to individuals’ health and well-being, and mental health disorders (e.g., depression) might peak later than the actual pandemic (5–7). Recently, China has embarked on a strategic initiative aimed at fostering applied and composite social talents through extensive vocational undergraduate education, which is intended to address the prevalent issue of structural unemployment, counteract the economic slowdown resulting from the COVID-19 pandemic, and enhance the competitiveness of its workforce within the global supply chain (8). The implementation of the strategy involves 32 vocational undergraduate institutions, and collectively enrolling approximately 41,000 students annually (9, 10). The majority of these students are recent high school graduates who either scored below average on their admission exams or hail from economically disadvantaged backgrounds. Beyond navigating the pressures associated with academic pursuits, many of them also face the premature challenge of career advancement, such as engaging in on-the-job training as early as first year of study, which early exposure to professional demands might exacerbate psychological concerns for these individuals (10, 11). Previous studies revealed vocational students experienced more anxiety, higher self-injurious behavior, and greater suicidal ideation than general students (12, 13). Thus, paying attention to the mental health (e.g., depressive symptoms) of vocational undergraduate students within college student cohorts appears necessary.

Depression is a widely recognized affective disorder characterized by decreased vitality and engagement in previously pleasurable activities, extreme fatigue, hopelessness, and loss of meaning in life (MIL; 14, 15). Clinically, individuals in life transition or with physical illness many reported meaning-centered concerns (16), and MIL has been closely associated with psychosocial factors that play a crucial role in health maintenance and recovery (17, 18). Within this context, the constructs of MIL, specifically the presence of meaning and the search for meaning, have emerged as promising predictors and concomitants of health (17, 19–21). The former relates to individuals’ understanding of their life experiences and pursuit of an overarching purpose, while the latter refers to the intensity and active pursuit of establishing or enriching their presence of meaning (16, 22). Both variables hold theoretical significance for mental and physical health (16), and previous research has shown links between low MIL and depressive symptoms. Disabato et al. (23) found in a longitudinal study that increased levels of MIL predicted decreased depressive symptoms at 3 and 6 months. Furthermore, an ERP study showed individual differences in MIL would forecast conflict responses when self-related health information was considered (17, 24). Therefore, exploring the role of MIL becomes particularly relevant when considering depression and it might also act as a buffer against depressive symptoms (25).

Meanwhile, Park and Folkman (26) formulated the meaning-making model in the setting of stress and trauma-related disorders. It proposes that after a stressful event, individuals continuously seek and assign meaning to their situations, relationships, behaviors, goals, and events. Then, by comparing and assessing the sense of meaning obtained from the situational stimuli with the self-existing beliefs, which would change and adjust according to the changing situation, i.e., situation meaning may change (20). Nevertheless, the perception of discrepancies (e.g., the situational meaning of event conflicts with one’s overall meaning) provokes distress in individuals. Usually, individuals strive to reduce the discrepancies through meaning-making (e.g., the constant search for meaning), and it is an effective coping strategy for dealing with stressors and also an adaptive response (18, 20). Moreover, in studies of meaning-making and psychological adjustment, Kernan and Lepore (27) found that the meaning-making process was related to more negative emotions and represented a distressing psychological state. Despite many factors that may influence meaning-making, some researchers believed that ties to family, friends, and teachers might be especially relevant and instrumental in psychological adjustment (2, 28, 29). Perceived social support (PSS), which constitutes a foundational protective element in the lives of adolescents, pertains to the subjective perceptions of emotional and tangible aid received from parents, friends, or significant individuals within one’s social circle. Extensive research endeavors have demonstrated that PSS plays a pivotal role in enhancing individuals’ positive psychological capital, encompassing hope, resilience, and MIL (2, 29, 30). Furthermore, it emerges as a crucial safeguard against the manifestation of depressive symptoms. Within the context of adolescents’ social networks, family holds an indispensable position, capable of fulfilling fundamental needs by providing emotional support and material resources. Friend support, on the other hand, facilitates the sharing of mutual experiences and positive interactions among adolescents, thereby mitigating the potential risk of depressive symptoms. School support, which represents a significant source of social reinforcement for adolescents during their school years, not only extends emotional and informative assistance to foster feelings of affection and the acquisition of problem-solving skills but also contributes to the alleviation of depressive symptoms (9, 30).

According to Beck’s cognitive model of mood disorders, it is postulated that stressful life events may precipitate or intensify depressive symptoms by fostering distorted cognitive processing. Within this framework, individuals predisposed to depression tend to interpret life events in a negative and self-deprecating light, ultimately cultivating feelings of hopelessness, helplessness, and worthlessness (10). Conversely, the main effect model of social support underscores the importance of positive experiences and social rewards derived from one’s social networks, encompassing family, schools, and communities. For vocational school students, PSS holds particular significance. Illustratively, female secondary vocational school students who perceive high levels of social support tend to exhibit heightened subjective well-being when confronted with stressful life events, which suggests that such support serves as a buffer against the adverse impact of stressful experiences, fostering resilience and enhancing overall well-being (9). As previously mentioned, both PSS and MIL were effective in mitigating the adverse effects of stress on individuals (9, 10). Notably, MIL not only alleviates the impact of stress but also exhibits growth and restorative effects. Evidence from a longitudinal survey revealed that trusting others to help with future needs correlated with a sense of deeper meaning (31). Simultaneously in the process, on the one hand, PSS could have a beneficial effect on mood through delivering positive regular experiences that boost personal perceptions and self-worth. On the other hand, it may also diminish the negative emotional impact of stressful life experiences, promoting more beneficial and less negative reactions to stressors by reducing the potential of negative assessments towards them (17, 26). Overall, although previous research has suggested that MIL and PSS strongly correlate with depressive symptoms (18, 32), they mostly used latent variable models to examine the relationships between MIL, PSS, and depressive symptoms. Such an approach might ignore relationships across items of psychological constructs and blur the significance of separate ones, which needs to be explored in a more microscopic perspective, i.e., using a network analysis approach to better understand the pathological route.

Network analysis has received increasing attention (33, 34), and the theory assumes that mental disorders are derived from the overall interconnectedness of their symptoms (35). Specifically, the appearance of one symptom is considered to increase the probability of the emergence of interrelated symptoms, in turn which could lead to episodes of illness. It differed from the latent variable model, which supposed unobservable latent variables resulting in observable symptoms (33). In a network, nodes represent symptoms, edges signify the relationship between symptoms (36), and centralities indicate node importance, referring to the connectivity of a node and its contribution to sustaining the disorder (37). Namely, centralities serve to evaluate the degree of interconnectedness relative to other nodes (38), and centrally activated symptoms might cause other symptoms to develop (39). Nevertheless, the goal of psychometric network analysis indeed extends beyond mere symptom relationships and encompasses understanding the associations among a broad range of measured psychological variables, including beliefs, traits, and, as applicable, constructs such as MIL and PSS. This analytical approach offers insights that can be more nuanced than traditional techniques, often outperforming them in determining the number of latent factors [e.g., (40)]. The primer on the network analysis of multivariate data in psychological science by Borsboom et al. provides a comprehensive overview of psychometric network analysis, detailing graphical models, estimation methods, and descriptive tools that facilitate such investigations (41). In numerous studies, direct analyses at both the symptom and latent factor levels have been conducted [e.g., (42)]. Within the frameworks of MIL and PSS, network analysis offers a refined comprehension of the intricate interconnections among the constituent elements of constructs. While total scores can be useful tools, they might not adequately encapsulate the full complexity of relationships (34). By scrutinizing the linkages at a more micro level, it is possible to elucidate how specific dimensions of MIL and PSS interact to influence depressive symptoms, thereby enriching the theoretical contributions to the existing scholarly discourse. Thus, it would be useful to use sophisticated interactive systems to examine the microelements between MIL, PSS, and depressive symptoms in more depth and to develop possible targeted interventions.

Hence, the present study constructed network structures related to MIL, PSS, and depressive symptoms for Chinese vocational undergraduate students after the pandemic. Specifically, we divided three subnetworks according to levels of depression and examined the features of each separate network structure and inter-network structure. The main objectives are threefold: (1) to explore associations between MIL, PSS, and depressive symptoms at different levels of depression, (2) to identify critical central nodes of subnetworks and variability between network structures, and (3) to identify critical bridge nodes for the transmission of MIL or PSS effects with depressive symptoms. Based on that, we seek to contribute theoretical insights for specific pathological pathways of MIL or PSS on depressive symptoms and to provide implications for clinical prevention or intervention.

## 2 Methods

### 2.1 Participants and procedure

We used convenience sampling for a survey study at a Northwestern vocational education university in China that was establishing a mental health center. To control the sampling error within 3% and consider statistical power (43), the sample size was computed by the formula: *n = Z^2^pq/d^2^*. *A* 95% confidence intervals (*CIs*; *Z* = 1.96), a population proportion of 50% (*p* = .50), and a 3% error rate (*d* = .03) was chosen, thus a minimum sample size *n* of 1067 was calculated. Data collection was performed in April through the *Wenjuanxing* website, a Chinese online survey platform. Informed consent was acquired from all participants, who were above 18 years old and were informed to participate voluntarily in advance. Additionally, the Human Ethics Committee of Ningxia University reviewed and approved the study (Project *No. NXU-23-051*). Consequently, 1520 questionnaires were returned, and any response time less than 120 seconds or a value selected in one or more scales was removed. Finally, 1367 valid questionnaires were obtained, with a valid rate of 89.9% and 611 (44.7%) were female (See **Table 1** for details).

**Table 1:** Sample demographic.

Characteristics		*N* (%)	Characteristics		*N* (%)
Gender	Male	755 (55.3)	Grade	One	881 (64.9)
	Female	611 (44.7)		Two	285 (21.0)
Race	Han Chinese	1083 (79.2)		Three	136 (10.0)
	Mongolian	217 (15.9)		Four	55 (4.1)
	Other	67 (4.9)	Birthplace	Urban	415 (30.4)
Only child		545 (39.9)		Rural	952 (69.6)
Loss		415 (30.4)			
Age		Mean (*SD*)			
		20.1 (1.6)			

*N* = 1367; loss represents the recent death of a family member in the past year.

### 2.2 Measures

#### 2.2.1 Meaning in life questionnaire

The MLQ-10 was developed by Steger et al. (22) to measure individuals’ sense of seeking and existential meaning. The 10-item questionnaire is scored on a 7-point Likert scale (1 = absolutely untrue; 7 = absolutely true). Scores indicate an individual’s level of MIL. It has been widely used in China and demonstrated good reliability and validity (44, 45). Within the MLQ-10, the sense of significance subscale, which measures the individual’s perception of importance and purpose in life, exhibited a reliability coefficient of.929 and McDonald’s omega of.946; the search for meaning subscale, which focuses on the individual’s active engagement in seeking meaning and purpose, demonstrated a reliability coefficient of.940 and McDonald’s omega of.953 in our study. Furthermore, confirmatory factor analysis (CFA) conducted on the two-factor model of the MLQ-10 scale showed a good model fit (RMSEA = .048, CFI= .979, TLI = .972, SRMR = .036).

#### 2.2.2 Perceived social support scale

The present study employed an adapted 12-item multidimensional scale of PSS to assess it from family, friends, and others (teachers or classmates); participants used a seven-point Likert scale to rate, with higher scores indicating greater PSS (46). The scale has been widely used in China and demonstrated high internal consistency (29, 47), with reliability coefficients of .961 (Cronbach’s alpha) and .966 (McDonald’s omega) in our study. Additionally, for the school dimension, a reliability coefficient of .942 and McDonald’s omega of .958 were obtained; for the family dimension, a reliability coefficient of .931 and McDonald’s omega of .951 were found; for the friend dimension, a reliability coefficient of .952 and McDonald’s omega of .965 were reported. Furthermore, CFA using a three-factor model indicated a good model fit for the PSS-12 (RMSEA = .043, CFI = .982, TLI = .976, SRMR = .019).

#### 2.2.3 Patient health questionnaire

The PHQ-9 is a self-administered screening tool used to assess depressive symptom severity, consisting of nine items based on DSM-IV criteria (48). Participants rate their mood over the past two weeks on a 4-point Likert scale. Scores range from 0-27, with higher scores indicating more severe symptoms. The Chinese version of the PHQ-9 demonstrated good reliability and validity (49). The Cronbach’s alpha was .916 and McDonald’s omega was .931 in our study, with CFA conducting a single-factor and showing a good model fit (RMSEA = .077, CFI = .944, TLI = .925, SRMR = .038).

### 2.3 Statistical analysis

Descriptive statistics and correlational analyses were conducted using SPSS 25.0. On the one hand, based on previous research (50), symptomatic individuals were categorized by PHQ-9 total scores into three separate groups, i.e., 1 ≤ score ≤ 4, 5 ≤ score ≤ 9, and score ≥ 10, named *minimal depressive symptoms*, *subthreshold depressive symptoms*, and *moderate/severe depressive symptoms* (48, 49). Notably, the participants who had no symptoms were excluded from that categorization and subsequent analyses. On the other hand, to proceed with the network analysis, PSS-12 was divided by total scores of three dimensions, named *family*, *friend*, and *school*. Additionally, depressive symptoms network analyses such as network estimation, bridge centrality estimation, and network comparison test (NCT) between the groups above were performed using version R 4.1.0.

#### 2.3.1 Network estimation

Initially, the selection operator in the *qgraph* package built the three depressive symptoms network structures (35, 51). However, the challenge of balancing the comprehensiveness of the network with the complexity of parameter estimation emerged, prompting careful consideration of node inclusion at either the item or subscale level. According to previous research [e.g., (42)], for the MLQ-10, we included items as network nodes to capture distinct aspects of MIL and gained a nuanced understanding of their interactions with depressive symptoms, providing a comprehensive picture; whereas for the PSS-12, we selected the three subscale scores as nodes, representing PSS from different sources, to reduce node count, simplify parameter estimation complexity, and still examine the overall structure of PSS, acknowledging varying weights and mechanisms of action among these support sources in influencing depressive symptoms. Given the potential for a massive number of parameters in each network (i.e., 22 nodes entail 253 parameters to estimate: 22 threshold parameters and 231 pairwise correlation parameters) might generate some false positive edges. Thus, we constructed a regularized partial correlation network to investigate links between parameters. Furthermore, for visualizing the networks, we adopted the *averageLayout* function, which generates a coherent layout by averaging the positions of different network structures (51), that is, the same nodes in different structures were fixed in the same position. Finally, in network estimation, centralities include node strength, betweenness, and closeness (35). Previous studies have considered betweenness and closeness as unstable (33, 34), thus we primarily relied on strength to gauge centrality.

#### 2.3.2 Bridge centrality estimation

We utilized the *networktools* package to identify critical nodes for cluster connectivity by computing the bridge strength based on edge weights from a given node to other clusters (36, 52). Nodes exhibiting a higher bridge strength are deemed to play a more significant role in activating nodes from the opposite clusters. To avoid confirmation bias in interpreting centrality statistics, we employed a stringent approach that involved blind 80th percentile cutoffs on both node bridge strength values when determining the centrality and bridge nodes (36).

#### 2.3.3 Network comparison

To evaluate potential differences in network structures among the three depressive symptom networks (e.g., network structure invariance, edge invariance, and global strength invariance), we employed NCT using the *NetworkComparisonTest* package (53). Initially, an omnibus test was conducted for each pair of networks to determine if all edges were identical. Subsequently, *post hoc* tests were conducted to quantify the number of different edges among each pair of networks (a total of 231 edges). To address multiple testing, the NCT utilized the Holm-Bonferroni method for *post hoc* corrections. Finally, to examine whether global strength estimates, which represent the sum of absolute edge values for each network, varied across the networks.

#### 2.3.4 Accuracy and stability test

We used the bootstrapping method from the *bootnet* package to assess the accuracy and robustness of network estimations (35). Specifically, we performed 1000 nonparametric bootstraps to obtain 95% *CIs* for each edge weight, thereby evaluating the accuracy of the estimated values. Higher *CIs* with overlapping edge weights indicate lower accuracy in the graphical representation. Additionally, we examined the centrality stability of the networks through correlation stability (*CS*) coefficients derived from 1000 case-dropping subsets bootstrap. These coefficients illustrate how the centrality index fluctuates as the proportion of the sample subset decreases (e.g., comparing the entire sample to only 40% of it). Greater fluctuations in centrality with decreasing sample proportions signify less stability. The *CS* coefficient indicates the maximum acceptable level of sample reduction, with a strong stability threshold set at 0.50 and a minimum threshold of no less than 0.25.

## 3 Results

### 3.1 Descriptive statistics

The three-factor model fitted well by performed CFA (χ^2^/*df* = 3.60, RMSEA = .044, CFI = .995, TLI = .992, SRMR = .020; details see **Supplementary Table A**), which demonstrated no serious common method variance. Prior to further analysis, we excluded 394 participants (28.82% of the total sample) who reported no depressive symptoms, as their inclusion might obscure the underlying patterns in the data. Following the recommendations of Fried (54), we examined the means and variability of all items (see **Supplementary Table B**) and reported descriptive statistics of the three depressive symptom network clusters (see **Table 2**), a higher score indicated a stronger propensity for the trait. For the remaining sample of 973 participants, the average total depressive symptoms score (*M* = 7.74, *SD* = 5.13) was slightly higher than adult normative samples (49, 55) and lower than the clinical sample in previous studies (49, 56). Furthermore, the moderate/severe depressive symptoms group had higher total depressive symptom scores than the clinical sample from previous studies. Additionally, the result of the multiple test indicated significant differences between the groups in depressive symptoms, with *F*(2, 970) = 1880.81, *p* <.001, which confirms the validity of the grouping.

**Table 2:** Descriptive statistics of the three depressive symptom network clusters.

Symptom abbreviation/cluster	Group A (*N* = 297)			Group B (*N* = 399)			Group C (*N* = 277)		
	*M*	*SD*	*r*	*M*	*SD*	*r*	*M*	*SD*	*r*
Sense of significance	23.23	5.57	-.133^*^	21.71	5.35	-.142^**^	20.59	6.40	-.151^*^
Search for meaning	24.25	5.96	-.121^*^	23.88	5.71	-.126^*^	24.01	6.25	-.001
School	21.30	4.61	-.110	18.84	5.50	-.149^**^	18.12	5.51	-.069
Family	21.97	4.61	-.162^**^	19.75	5.48	-.194^**^	18.58	5.23	-.158^**^
Friend	22.08	4.35	-.105	20.02	4.99	-.143^**^	19.35	5.22	-.128^*^
Depression	2.47	1.15	—	7.11	1.35	—	14.29	3.86	—

*N* = 973; ^*^ *p* < 0.05, ^**^ *p* < 0.01; *r* refers to the Pearson correlation coefficient between clusters and depressive symptoms; Groups A, B, and C are characterized by minimal, subthreshold, and severe depressive symptoms, respectively. *Mean* is the dimension or scale total score divided by the corresponding items.

### 3.2 Network estimation

**Figure 1:**
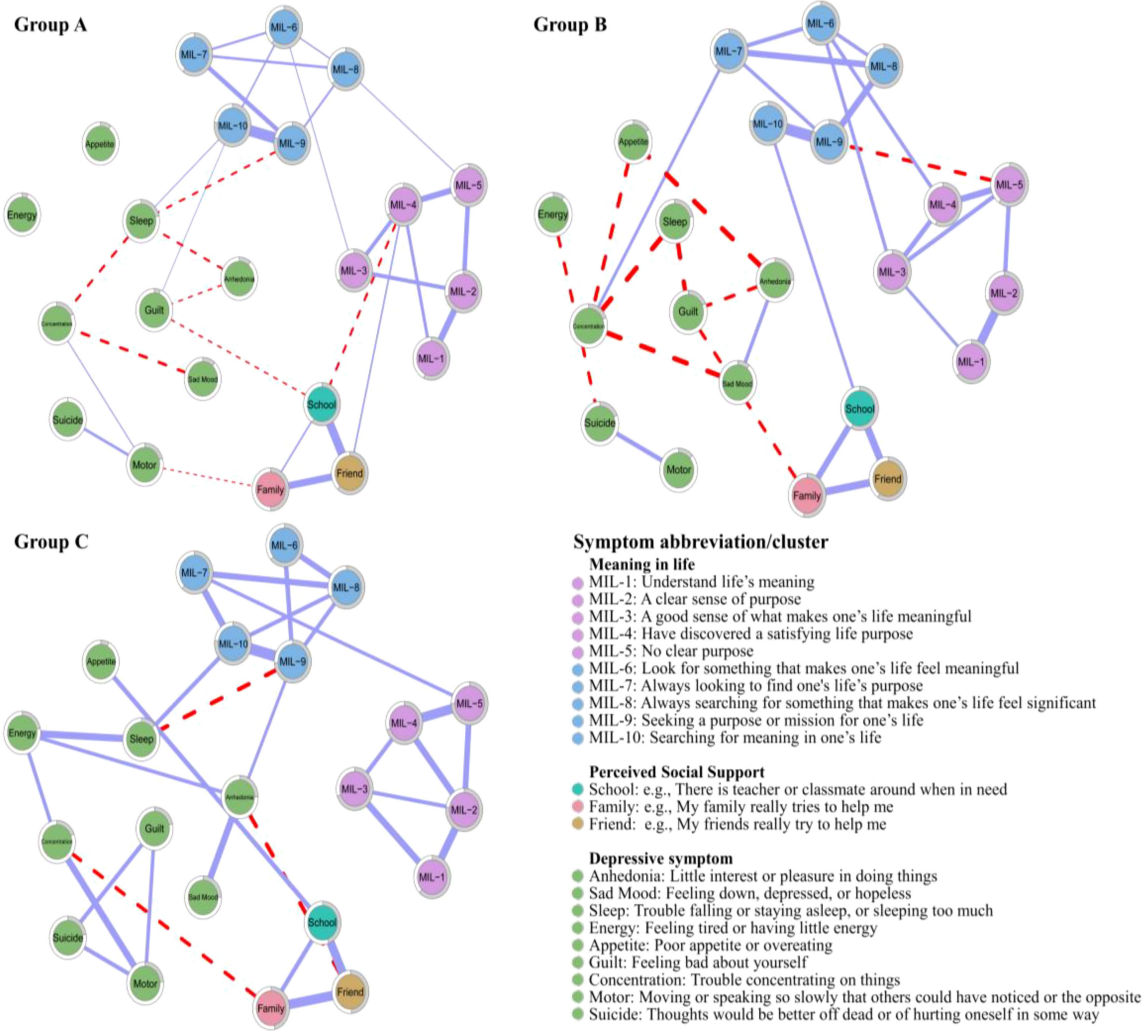
Network structures for three groups (cut value = 0.03, minimum value =0.15); the grey area of the nodal ring depicts undirected predictability (57).

**Figure 2:**
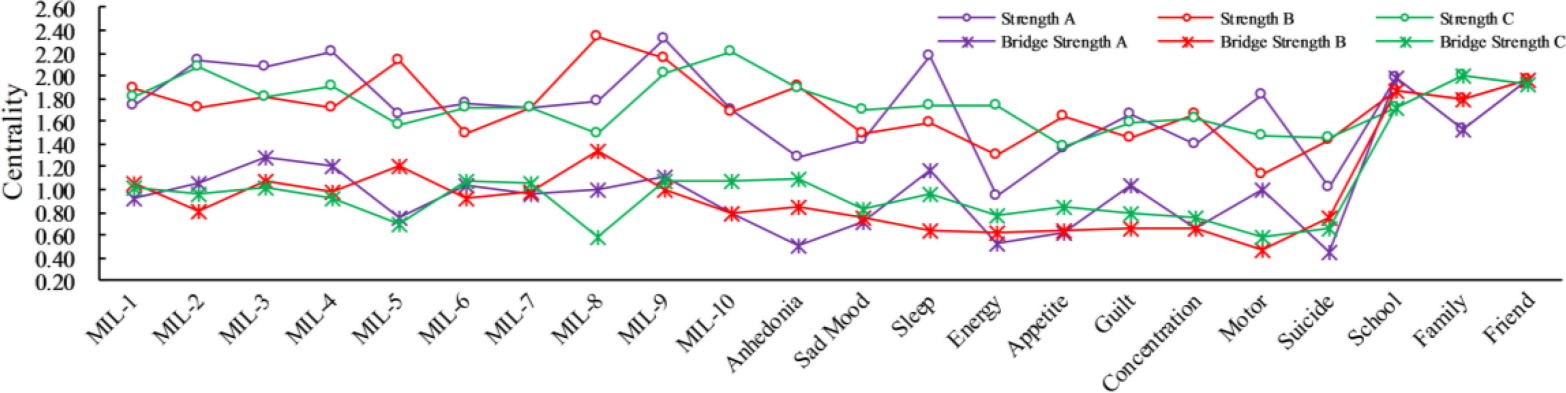
Strength and bridge strength centralities are node centrality weights (36, 58), see **Figure 1** or **Supplementary Table B** for symptom descriptions and names.

**Figure 1** depicts regularized partial correlation networks for three groups. Overall, the three networks with *sense of significance*, *search for meaning*, and *perceived social support* clustered more tightly together in each featured many consistent edges. From A to B and then to C, although depressive symptoms varied dramatically between groups, the connectivity was increasing, i.e., depressive symptoms were strengthening (See **Figure 2** for strength centrality). Meanwhile, *motor* and *suicide* had consistently stronger connectivity within the three groups. Within each group, there were 22 items and 231 edges, and Group A and B observed more negative edges than positive edges, while Group C did exactly the opposite. For more details on edge weights, see **Supplementary Table C**.

### 3.3 Network inference

#### 3.3.1 Bridge symptoms

After carefully considering the trends in the distribution of the three network structures, we selected the top 20% of nodes in terms of corresponding centrality scores for discussion, and the ratio produced an acceptable balance between specificity and sensitivity, similar to previous studies (36). The strength and bridge strength values are shown in **Figure 2** and **Supplementary Table D**. First, in Group A, some items showed a higher strength within aggregated clusters, e.g., items *MIL-9* (*strength value* = 2.322), *MIL-4* (*strength value* = 1.202), *MIL-2* (*strength value* = 2.136), *sleep* (*strength value* = 2.171), *motor* (*strength value* = 1.829), and *school* (*strength value* = 1.979), which indicated these items were the more cored nodes. Meanwhile, bridge nodes were *MIL-3* (*bridge strength value* = 1.281), *MIL-4* (*bridge strength value* = 1.213), *sleep* (*bridge strength value* = 1.170), *guilt* (*bridge strength value* = 1.043), and *school* (*bridge strength value* = 1.979). Second, in Group B, *MIL-8* (*strength value* = 2.338), *MIL-9* (*strength value* = 2.152), *MIL-5* (*strength value* = 2.134), *anhedonia* (*strength value* = 1.913), *concentration* (*strength value* = 1.658), and *friend* (*strength value* = 1.973) were the top cored nodes. = 1.206), *anhedonia* (*bridge strength value* = 0.853), *suicide* (*bridge strength value* = 0.754), and *friend* (*bridge strength value* = 1.973). Finally, in Group C, the top cored nodes were *MIL-10* (*strength value* = 2.208), *MIL-2* (*strength value* = 2.086), *MIL-9* (*strength value* = 2.023), *anhedonia* (*strength value* = 1.900), *sleep* (*strength value* = 1.742), and *family* (*strength value* = 2.008); bridge nodes were MIL-9 (*bridge strength value* = 1.084), *MIL-7* (*bridge strength value* = 1.058), *anhedonia* (*bridge strength value* = 1.099), *sleep* (*bridge strength value* = 0.956), and *family* (*bridge strength value* = 2.008).

#### 3.3.2 Network comparisons

To obtain the similarity coefficient of the three networks, we correlated the edge weights of each pair of networks. Based on Spearman’s correlation, the networks showed a moderate correlation. Furthermore, we also tested the overall strength estimates (i.e., connectivity) and compared differences in the network structure and centrality. The global strength values of the three groups were 6.44 for Group A, 7.88 for Group B, and 7.01 for Group C. The network invariance test showed no significant differences between pairwise comparisons of the two groups, yet the centrality test indicated significant differences between the groups (see **Table 3** for details).

**Table 3:** NCT indexes across three groups.

Comparison	Similarity test				Global strength invariance test		Network invariance test		Centrality test	
	Edge weights	Strength	Bridge strength	Predictability	*S*	*p*	*M*	*p*	*C*	*p*
Group B ↔ Group A	.415^**^	.348	.578^**^	.879^**^	1.441	.02	.178	.86	-.281	.01
Group C ↔ Group B	.243^**^	.373	.548^**^	.860^**^	0.870	.10	.230	.39	.281	.02
Group C ↔ Group A	.280^**^	.431^*^	.517^*^	.947^**^	0.571	.08	.218	.65	.000	.02

^*^ *p* < 0.05, ^**^ *p* < 0.01; similarity was calculated using Spearman correlation, and all tests were two-tailed.

### 3.4 Accuracy of the networks

The relatively narrow 95% *CIs* of the bootstrap indicate that the edges of networks would be judged accurately (see **Supplementary Figures S1**–**S3**), and we performed bootstrap difference tests for edge weights and node strength of the three sets of networks separately (see **Supplementary Figures S4**–**S9**). The CS coefficients for edge, strength, and bridge strength in Group A (.751/.751/.751), Group B (.749/.749/.749), and Group C (.751/.751/.751) were > 0.25, which meet the cutoff score suggested by Epskamp et al. (35). In addition, the correlation stability coefficients for the edge, node strength, and bridge strength decreased smoothly in line with the reducing proportion of sampled cases (see **Supplementary Figures S10**–**S12** for details). It demonstrated that the centrality indices of edge, node strength, and bridge strength were sufficiently stable.

## 4 Discussion

The economic repercussions of the pandemic have emphasized the notion of “scarring effects”, where lasting impressions are indelibly etched onto economies. While economies endeavor to revert to their pre-pandemic states, individuals likewise confront prolonged repercussions stemming from the event (59). Existing research suggested that MIL and PSS relieve depressive symptoms. We used network analysis, specifically through node centrality, bridge strength centrality, and NCT to further explore the complicated interrelationships between MIL and PSS with specific depressive symptoms in vocational undergraduate students. First, we found that MIL, PSS, and depressive clusters were linked within each network structure, which was similar to previous studies using multivariate models (18, 23, 29), demonstrating that all three variables were related both in latent variable models and network analyses. Additionally, on one hand, within each network structure, intracluster connections were tighter than intercluster connections, which was similar to previous findings that symptom clusters converged better (34). On the other hand, across the three networks, the clusters of MIL and PSS did not change visibly in connectivity, whereas the depressive cluster changed more dramatically from *minimal* → *subthreshold* → *moderate/severe*, with reinforced connectivity, i.e., enhanced symptoms. Notably, there was a consistently strong concatenation of *suicide* and *motor*, which demonstrates the persistence of symptoms and suggests that the existing depression diagnosis might be inappropriate to use total scores as classification criteria. Thus, potential hazards could vary across symptoms and perhaps more attention needs to be paid to individuals with suicidal tendency symptom (60).

Second, we found that *anhedonia* and *concentration* were the two strongest symptom nodes for moderate/severe depressive symptoms in terms of central and bridge symptoms. The node strength difference significance test also showed that both nodes were significantly stronger than the others in the same network group. It was similar to previous studies (50), yet different from some studies [e.g., (61)] that considered *sad mood* as the most central node of the network. Furthermore, *sleep*, *anhedonia*, and *sleep* played bridge node roles in Group A, Group B, and Group C, respectively, for depressive symptom clusters. In Asian populations, individuals tend to report their distress as somatic symptoms (e.g., sleeplessness) rather than as emotional symptoms (50, 62), which was related to their epistemology of disease and a stigma tendency (63). According to a systematic review, the ‘typical’ presentation of depression in primary care is dominated by somatic complaints, with more than 50% of depressed clients reporting only somatic complaints (64).

Finally, from NCT, global strength invariance and network invariance didn’t vary much, whereas the centrality test showed that Group B changed more and had the highest global strength, which may indicate that depressive symptoms scoring 5 - 9 individuals are not non-depressed, rather a subthreshold, i.e., other depression (48). Although the PHQ-9 design is precisely derived from the nine diagnostic criteria for DSM-IV depression, it has fewer diagnostic items, thus the scored segment requires caution for more specific clinical diagnosis, such as using the Hamilton tool (48, 49). Simultaneously, the sense of significance decreases from Group A → Group B → Group C, with the search for meaning (MIL-6 and MIL-9 in Group C) emerging as bridge nodes, suggesting that meaning-making may be an effective strategy for coping and adapting to stressors and depression (20), and research on cognitive behavioral therapy outcomes could attribute significant clinical improvements in depressive symptoms to that (32). Additionally, we conducted our research in a Confucian context, contrary to Western culture, in which a high level of the pursuit of meaning is conducive to good mental health (62), and Breitbart (65) has developed a group psychotherapy for patients called “meaning-centered psychotherapy” that may also be suitable for vocational undergraduate students curricula aimed at mental health education in a Confucian cultural context. Overall, numerous studies have shown that meaning in life is related to mental health (17, 18), and it seems worthwhile to seek more effective ways to encourage and support young people’s search for meaning (66), a process that cannot ignore the role of *school* → *friend* → *family* at corresponding stages of depressive symptoms.

The present study’s findings have practical implications that could guide both educational interventions and mental health support programs for vocational undergraduates. When symptoms are interconnected and constantly reinforce each other synergistically, relationships within symptoms in turn become self-sustaining, such a self-reinforcing feedback loop is assumed to be a beginning and continuation of mental health problems, and the intensity of symptoms within the network predicts future psychological health concerns that may escalate into a crisis (35, 39). Thus, central nodes are considered potentially critical for therapeutic interventions. In our study, *anhedonia* and *concentration* are the central nodes to focus on for subthreshold depressive symptoms, while *anhedonia* and *sleep* are the ones that require attention for moderate/severe depressive symptoms. Turning complex psychotherapies into intervention ideas with more bite is a challenge, especially given the emphasis on personal phenomenology in existential approaches. Cognitive Therapy based on mindfulness, Narrative Therapy, Behavioral Activation Therapy, and Person-Centered Psychotherapy may increase MIL, as they correspond to teaching individuals skills to manage their consciousness, assisting them to integrate past, present and future time, enabling them to identify and engage in activities that allow them to act in the world, and helping them to develop authenticity and other mature perspectives about themselves (16, 18). An earlier meta-analysis showed that regardless of the formal treatment direction deployed, the estimated effect size for meaning-based practices was a moderate effect [*β* = .38; (16)]. The Chinese emphasized the interconnectedness of mental, physical, and social life, both at the personal and family levels (63).

While for vocational undergraduates, school is an invaluable resource for guiding students to understand meaning in their lives and incorporating it into a positive education curriculum, e.g., explicitly urging students to set attainable goals in daily life, use mindfulness practices, address self-worth, and concentrate on creating positive interpersonal relationships (66). Considering the high level of distress in the youth today and the fact that the search for meaning is a dysfunctional process with the potential to lead to depressive symptoms and discomfort (32, 67–69). However, the Chinese are deeply influenced by Confucianism, an interdependent culture in which the search for meaning has a positive effect as they consider the world in a holistic or dialectical way and the famous doctrine as “Heaven maintains vigor through movements, a gentleman should constantly strive for self-perfection” inspires youth to adopt positive attitudes toward life, to affirm and pursue life regardless of their current life situation (62) and harmful effects of the search for meaning are also reduced by the heightened presence of meaning (18). It is noteworthy that our research primarily focuses on Chinese vocational university students, acknowledging the pivotal role that cultural and societal factors play in shaping the experiences and expressions of mental health issues. Chinese culture strongly emphasizes collectivism, social harmony, and family bonds (30), which could significantly influence how individuals perceive and cope with stressful events and subsequent depressive symptoms. On the one hand, the emphasis on filial piety, familial duties, and the centrality of family life in Chinese culture may influence how individuals perceive and respond to stressful events, such as the loss of a family member. Cultural nuances might lead to unique interpretations and reactions to these events, differing from those observed in Western cultures (11, 30). The interplay between cultural norms and individual responses to such events is a crucial aspect to consider. On the other hand, the collectivist nature of Chinese society often prioritizes the well-being of the family or community over personal mental health (10, 59). This cultural orientation could affect how individuals interpret and communicate their negative experiences, influencing their willingness to seek help and support. Understanding the cultural dynamics of collectivism and individualism is crucial when interpreting our findings (9, 30). Anyway, the present study enriches our understanding of the complexities of mental health and informs more culturally sensitive interventions and support programs. Consequently, a strategy that incorporates periodic screening to identify vocational undergraduate students’ psychological features, along with flexible interventions and curricular changes, would be more effective in addressing their mental health needs.

Although our study yielded novel findings, several limitations remain to be considered. First, community samples may have low levels of psychopathology (33, 54). The practice of selecting participants based solely on the sum of depressive symptoms criteria might introduce Berkson’s bias. Furthermore, despite the data from the three groups being normally distributed, the selection method might distort the relationships among variables. Therefore, future studies recruiting participants based on genetic and environmental risk factors may yield more reliable conclusions (70). Second, the uncertainty associated with cross-sectional sampling and variability in sample composition is another concern. Despite our intentional increase in participants, sampling variations and power limitations should not be ignored (71). Specifically, given the complexity of the current multi-node network model, the statistical power to estimate networks within subgroups of depressive symptoms may be constrained, which would likely affect the stability and reliability of individual nodes, even with using regularization methods and bootstrapping procedures in place to ensure accuracy and stability. Moreover, the cross-sectional data limits the capability to draw robust causal inferences (59). Additionally, all participants were recruited from a university in China, which might affect the applicability of the findings to a broader young cohort. Finally, experimental appreciation might be another fundamental contributor to MIL (72). Future relevant studies could further explore its role in depressive symptoms, providing additional insights into the complex relationship.

## 5 Conclusion

This study used network analysis for the first time to explore interactions between MIL, PSS, and depressive symptoms in vocational undergraduate students at different levels of depression. The results elucidated concrete pathways for the interaction of MIL or PSS on depressive symptoms. Specifically, we identified potential central and bridge symptom nodes at different levels of depression. Moreover, NCT showed that network structures and strengths were largely homogeneous, yet network centrality was inconsistent. Our findings could provide specific targets for pathways to effectively prevent and intervene in depressive symptoms treatment.

## Data Availability

The raw data supporting the conclusions of this article will be made available by the authors, without undue reservation.

## References

[1] Bao Y Li L Guan Y Wang W Liu Y Wang P. Prevalence and associated positive psychological variables of anxiety and depression among patients with central nervous system tumors in China: A cross-sectional study. Psycho-Oncology. (2017) 26:262–9. doi: 10.1002/pon.4128 27072749

[2] Rapee RM Oar EL Johnco CJ Forbes MK Fardouly J Magson NR. Adolescent development and risk for the onset of social-emotional disorders: A review and conceptual model. Behav Res Ther. (2019) 123:103501. doi: 10.1016/j.brat.2019.103501 31733812

[3] World Health Organization. Depression and other common mental disorders: Global health estimates. (2017). Available online at: https://apps.who.int/iris/ (Accessed September 12, 2024).

[4] Salari N Hosseinian-Far A Jalali R Vaisi-Raygani A Rasoulpoor S Mohammadi M. Prevalence of stress, anxiety, depression among the general population during the COVID-19 pandemic: A systematic review and meta-analysis. Globalization Health. (2020) 16:57–67. doi: 10.1186/s12992-020-00589-w 32631403 PMC7338126

[5] Santomauro DF Herrera AMM Shadid J Zheng P Ashbaugh C Pigott DM. Global prevalence and burden of depressive and anxiety disorders in 204 countries and territories in 2020 due to the COVID-19 pandemic. Lancet. (2021) 398:1700–12. doi: 10.1016/S0140-6736(21)02143-7 PMC8500697 34634250

[6] Xiao Y Du N Li Y. Mental health services in China: Challenges in the context of COVID-19. Asian J Psychiatry. (2023) 80:103348. doi: 10.1016/j.ajp.2022.103348 PMC9684096 36444825

[7] Sher L. The impact of the COVID-19 pandemic on suicide rates. Qjm: Int J Med. (2020) 113:707–12. doi: 10.1093/qjmed/hcaa202 PMC7313777 32539153

[8] China’s Ministry of Education. The guidelines for deepening the reform of modern vocational education system construction (2023). Available online at: http://www.gov.cn/gongbao/content/2023/content_5736711.htm (Accessed September 16, 2024).

[9] He Y Zeng Q Zhang M. The mediating roles of future work self and hope on the association between perceived social support and depressive symptoms among Chinese vocational school students: A cross-sectional study. Psychol Res Behav Manage. (2023) 16:2125–36. doi: 10.2147/PRBM.S414356 PMC10275316 37334406

[10] Zhang S Ding F Sun Y Jing Z Li N. Negative life events on depression of vocational undergraduates in the partial least squares structural equation modeling approach perspective: A mediated moderation model. Behav Sci. (2023) 13:895–908. doi: 10.3390/bs13110895 37998642 PMC10669152

[11] Zhang H Sang Z Chan DK-S Teng F Liu M Yu S. Sources of meaning in life among Chinese university students. J Happiness Studies: Interdiscip Forum Subjective Well-Being. (2016) 17:1473–92. doi: 10.1007/s10902-015-9653-5

[12] Dalen JD. The association between school class composition and suicidal ideation in late adolescence: Findings from the young-hunt 3 study. Child Adolesc Psychiatry Ment Health. (2012) 6:37. doi: 10.1186/1753-2000-6-37 23186517 PMC3539984

[13] Horvath LO Balint M Ferenczi-Dallos G Farkas L Gadoros J Gyori D. Direct self-injurious behavior (D-SIB) and life events among vocational school and high school students. Int J Environ Res Public Health. (2018) 15:10686. doi: 10.3390/ijerph15061068 PMC6025121 29795028

[14] van Borkulo C Boschloo L Borsboom D Penninx BWJH Waldorp LJ Schoevers RA. Association of symptom network structure with the course of depression. JAMA Psychiatry. (2015) 72:1219–26. doi: 10.1001/jamapsychiatry.2015.2079 26561400

[15] Wolk CB Carper MM Kendall PC Olino TM Marcus SC Beidas RS. Pathways to anxiety-depression comorbidity: A longitudinal examination of childhood anxiety disorders. Depression Anxiety. (2016) 33:978–86. doi: 10.1002/da.22544 PMC5050087 27433887

[16] Vos J Vitali D. The effects of psychological meaning-centered therapies on quality of life and psychological stress: A meta-analysis. Palliative Supportive Care. (2018) 16:608–32. doi: 10.1017/S1478951517000931 30246682

[17] King LA Hicks JA. The science of meaning in life. Annu Rev Psychol. (2021) 72:561–84. doi: 10.1146/annurev-psych-072420-122921 32898466

[18] Steger M. Making meaning in life: A thematic review of successful experimental psychological and psychotherapeutic interventions. In: *Proceedings of the meaning in life international conference 2022 - Cultivating, promoting, and enhancing meaning in life across cultures and life span* (5-20). Dordrecht: Atlantis Press (2022). https://www.atlantis-press.com/proceedings/mil-22/125980408.

[19] FioRito TA Routledge C Jackson J. Meaning-motivated community action: The need for meaning and prosocial goals and behavior. Pers Individ Dif. (2021) 171:110462. doi: 10.1016/j.paid.2020.110462

[20] Park J Baumeister RF. Meaning in life and adjustment to daily stressors. J Positive Psychol. (2017) 12:333–41. doi: 10.1080/17439760.2016.1209542

[21] Park N Park M Peterson C. When is the search for meaning related to life satisfaction? Appl Psychology: Health Well-being. (2010) 2:1–13. doi: 10.1111/j.1758-0854.2009.01024.x

[22] Steger MF Frazier P Oishi S Kaler M. The meaning in life questionnaire: Assessing the presence of and search for meaning in life. J Couns Psychol. (2006) 53:80–93. doi: 10.1037/0022-0167.53.1.80

[23] Disabato DJ Kashdan TB Short JL Jarden A. What predicts positive life events that influence the course of depression? A longitudinal examination of gratitude and meaning in life. Cogn Ther Res. (2017) 41:444–58. doi: 10.1007/s10608-016-9785-x

[24] Kang Y Strecher VJ Kim E Falk EB. Purpose in life and conflict-related neural responses during health decision-making. Health Psychol. (2019) 38:545–52. doi: 10.1037/hea0000729 PMC7233478 31008647

[25] Guerrero-Torrelles M Monforte-Royo C Rodriguez-Prat A Porta-Sales J Balaguer A. Understanding meaning in life interventions in patients with advanced disease: A systematic review and realist synthesis. Palliative Med. (2017) 31:798–813. doi: 10.1177/0269216316685235 28498025

[26] Park CL Folkman S. Meaning in the context of stress and coping. Rev Gen Psychol. (1997) 1:115–44. doi: 10.1037/1089-2680.1.2.115

[27] Kernan WD Lepore SJ. Searching for and making meaning after breast cancer: Prevalence, patterns, and negative affect. Soc Sci Med. (2009) 68:1176–82. doi: 10.1016/j.socscimed.2008.12.038 19157667

[28] Lambert NM Stillman TF Hicks JA Kamble S Baumeister RF Fincham FD. To belong is to matter: Sense of belonging enhances meaning in life. Pers Soc Psychol Bull. (2013) 39:1418–27. doi: 10.1177/0146167213499186 23950557

[29] Liu C Huang N Fu M Zhang H Feng XL Guo J. Relationship between risk perception, social support, and mental health among general Chinese population during the COVID-19 pandemic. Risk Manage Healthcare Policy. (2021) 14:1843–53. doi: 10.2147/RMHP.S302521 PMC8114362 33994815

[30] Lyu C. Relations between perceived social support and prosocial behavior among Chinese college students during online learning: Testing mediation and moderation models of meaning in life. Heliyon. (2024) 10:e37677. doi: 10.1016/j.heliyon.2024.e37677 39323776 PMC11422598

[31] Krause N. Longitudinal study of social support and meaning in life. Psychol Aging. (2007) 22:456–69. doi: 10.1037/0882-7974.22.3.456 17874947

[32] Marco JH Alonso S Banos R. Meaning-making as a mediator of anxiety and depression reduction during cognitive behavioral therapy intervention in participants with adjustment disorders. Clin Psychol Psychother. (2021) 28:325–33. doi: 10.1002/cpp.2506 32881109

[33] Bringmann LF Albers C Bockting C Borsboom D Ceulemans E Cramer A. Psychopathological networks: Theory, methods, and practice. Behav Res Ther. (2022) 149:104011. doi: 10.1016/j.brat.2021.104011 34998034

[34] McNally RJ. Network analysis of psychopathology: Controversies and challenges. Annu Rev Clin Psychol. (2021) 17:31–53. doi: 10.1146/annurev-clinpsy-081219-092850 33228401

[35] Epskamp S Borsboom D Fried EI. Estimating psychological networks and their accuracy: A tutorial paper. Behav Res Methods. (2018) 50:195–212. doi: 10.3758/s13428-017-0862-1 28342071 PMC5809547

[36] Jones PJ Ma R McNally RJ. Bridge centrality: A network approach to understanding comorbidity. Multivariate Behav Res. (2021) 56:353–67. doi: 10.1080/00273171.2019.1614898 31179765

[37] Hofmann SG Curtiss J McNally RJ. A complex network perspective on clinical science. Perspect psychol Sci. (2016) 11:597–605. doi: 10.1177/1745691616639283 27694457 PMC5119747

[38] Kalisch R Cramer AO Binder H Fritz J Leertouwer I Lunansky G. Deconstructing and reconstructing resilience: A dynamic network approach. Perspect psychol Sci. (2019) 14:765–77. doi: 10.1177/1745691619855637 31365841

[39] Borsboom D. Reflections on an emerging new science of mental disorders. Behav Res Ther. (2022) 156:104127. doi: 10.1016/j.brat.2022.104127 35934488

[40] Golino H Shi D Christensen AP Garrido LE Nieto MD Sadana R. Investigating the performance of exploratory graph analysis and traditional techniques to identify the number of latent factors: A simulation and tutorial. psychol Methods. (2020) 25:292–320. doi: 10.1037/met0000255 32191105 PMC7244378

[41] Malhi GS Mann JJ. Depression. Lancet. (2018) 392:2299–312. doi: 10.1016/S0140-6736(18)31948-2 30396512

[42] Jing Z Ding F. Interaction between anxiety symptoms and decreased meaning in life: One possible pathway linking childhood trauma and depression-evidence from the network analysis. J Affect Disord. (2024) 355:440–9. doi: 10.1016/j.jad.2024.04.009 38580034

[43] Hayes AF Scharkow M. The relative trustworthiness of inferential tests of the indirect effect in statistical mediation analysis: Does method really matter? psychol Sci. (2013) 24:1918–27. doi: 10.1177/0956797613480187 23955356

[44] Ding FQ Tian XY Chen L Wang XF. The relationship between physical health and fear of death in rural residents: The mediation effect of meaning in life and mental health. Death Stud. (2022) 46:148–56. doi: 10.1080/07481187.2020.1723741 32027226

[45] Zhang S Tang Y Yong S. The influence of gratitude on pre-service teachers’ career goal self-efficacy: Chained intermediary analysis of meaning in life and career calling. Front Psychol. (2022) 13:843276. doi: 10.3389/fpsyg.2022.843276 35967650 PMC9367964

[46] Guan NC Seng LH Ann AYH Hui KO. Factorial validity and reliability of the Malaysian simplified Chinese version of multidimensional scale of perceived social support (MSPSS-SCV) among a group of university students. Asia-Pacific J Public Health. (2015) 27:225–31. doi: 10.1177/1010539513477684 23449622

[47] Sun S Goldberg SB Lin D Qiao S Operario D. Psychiatric symptoms, risk, and protective factors among university students in quarantine during the COVID-19 pandemic in China. Globalization Health. (2021) 17(1):15. doi: 10.1186/s12992-021-00663-x 33494769 PMC7829620

[48] Kroenke K Spitzer RL Williams J. The PHQ-9-validity of a brief depression severity measure. J Gen Internal Med. (2001) 16:606–13. doi: 10.1046/j.1525-1497.2001.016009606.x PMC1495268 11556941

[49] Horwitz AGG Zhao Z Sen S. Peak-end bias in retrospective recall of depressive symptoms on the PHQ-9. psychol Assess. (2023) 35:378–81. doi: 10.1037/pas0001219 PMC10052790 36757996

[50] Yang Y Zhang S Yang BX Li W Sha S Jia F. Mapping network connectivity among symptoms of depression and pain in Wuhan residents during the late-stage of the COVID-19 pandemic. Front Psychiatry. (2022) 13:814790. doi: 10.3389/fpsyt.2022.814790 35370830 PMC8968182

[51] Epskamp S Cramer AOJ Waldorp LJ Schmittmann VD Borsboom D. qgraph: Network visualizations of relationships in psychometric data. J Stat Software. (2012) 48:1–18. doi: 10.18637/jss.v048.i04

[52] Heeren A Jones PJ McNally RJ. Mapping network connectivity among symptoms of social anxiety and comorbid depression in people with social anxiety disorder. J Affect Disord. (2018) 228:75–82. doi: 10.1016/j.jad.2017.12.003 29232567

[53] van Borkulo CD van Bork R Boschloo L Kossakowski JJ Tio P Schoevers RA. Comparing network structures on three aspects: A permutation test. psychol Methods. (2022) 28:1273–85. doi: 10.1037/met0000476 35404628

[54] Fried EI. Tutorial: How to review psychopathology network papers (2017). Available online at: https://psych-networks.com/how-to-review-network-papers/ (Accessed October 18, 2024).

[55] Kocalevent R Hinz A Braehler E. Standardization of the depression screener patient health questionnaire (PHQ-9) in the general population. Gen Hosp Psychiatry. (2013) 35:551–5. doi: 10.1016/j.genhosppsych.2013.04.006 23664569

[56] Sun Y Fu Z Bo Q Mao Z Ma X Wang C. The reliability and validity of PHQ-9 in patients with major depressive disorder in psychiatric hospital. BMC Psychiatry. (2020) 20:4741. doi: 10.1186/s12888-020-02885-6 PMC7525967 32993604

[57] Haslbeck JMB Waldorp LJ. mgm: Estimating time-varying mixed graphical models in high-dimensional data. J Stat Software. (2020) 93:1–46. doi: 10.18637/jss.v093.i08

[58] Opsahl T Agneessens F Skvoretz J. Node centrality in weighted networks: Generalizing degree and shortest paths. Soc Networks. (2010) 32:245–51. doi: 10.1016/j.socnet.2010.03.006

[59] Zhang S Ding F Chen J. Comorbidity of anxiety and depression disorder among clinical referral patients: A longitudinal study based on network analysis. Curr Psychol. (2024) 43:20655–67. doi: 10.1007/s12144-024-05856-2

[60] Shin D Kim K Lee S Lee C Bae YS Cho WI. Detection of depression and suicide risk based on text from clinical interviews using machine learning: Possibility of a new objective diagnostic marker. Front Psychiatry. (2022) 13:801301. doi: 10.3389/fpsyt.2022.801301 35686182 PMC9170939

[61] Junghänel M Thöne A Dose C Breuer D Görtz-Dorten A Döpfner M. Conceptualizing anxiety and depression in children and adolescents: A latent factor and network analysis. Curr Psychol. (2023) 43:1248–63. doi: 10.1007/s12144-023-04321-w

[62] Huo J Wang X Ge Y Wang Y Hu X Liu M. Chinese college students’ ability to recognize facial expressions based on their meaning-in-life profiles: An eye-tracking study. J Pers. (2021) 89:514–30. doi: 10.1111/jopy.12596 32996593

[63] Ahmad F Maule C Wang J Fung WLA. Symptoms and experience of depression among Chinese communities in the West: A scoping review. Harvard Rev Psychiatry. (2018) 26:340–51. doi: 10.1097/HRP.0000000000000202 30407233

[64] Bair MJ Robinson RL Katon W Kroenke K. Depression and pain comorbidity: A literature review. Arch Internal Med. (2003) 163:2433–45. doi: 10.1001/archinte.163.20.2433 14609780

[65] Breitbart W. Creating your soul in every moment: Meaning, creativity, and attitude. Palliative Supportive Care. (2015) 13:1139–40. doi: 10.1017/S1478951515001133 PMC5490659 26399747

[66] Steger MF O Donnell MB Morse JL. Helping students find their way to meaning: Meaning and purpose in education. In: Kern ML Wehmeyer ML, editors. The Palgrave Handbook of Positive Education. Berlin: Springer International Publishing (2021). p. 551–79. https://link.springer.com/chapter/10.1007/978-3-030-64537-3_22.

[67] Burrow AL O’Dell AC Hill PL. Profiles of a developmental asset: Youth purpose as a context for hope and well-being. J Youth Adolescence. (2010) 39:1265–73. doi: 10.1007/s10964-009-9481-1 19937095

[68] Cohen K Cairns D. Is searching for meaning in life associated with reduced subjective well-being? Confirmation and possible moderators. J Happiness Stud. (2012) 13:313–31. doi: 10.1007/s10902-011-9265-7

[69] Lopez FG Ramos K Nisenbaum M Thind N Ortiz-Rodriguez T. Predicting the presence and search for life meaning: Test of an attachment theory-driven model. J Happiness Stud. (2015) 16:103–16. doi: 10.1007/s10902-013-9498-8

[70] de Ron J Fried EI Epskamp S. Psychological networks in clinical populations: Investigating the consequences of Berkson’s bias. Psychol Med. (2021) 51:168–76. doi: 10.1017/S0033291719003209 31796131

[71] Hoekstra RHA Epskamp S Borsboom D. Heterogeneity in individual network analysis: Reality or illusion? Multivariate Behav Res. (2022) 58:762–86. doi: 10.1080/00273171.2022.2128020 36318496

[72] Kim J Holte P Martela F Shanahan C Li Z Zhang H. Experiential appreciation as a pathway to meaning in life. Nat Hum Behav. (2022) 6:677. doi: 10.1038/s41562-021-01283-6 35145278

