# Supplementary material for: Network analysis of meaning in life, perceived social support, and depressive symptoms among vocational undergraduate students
Source: Front Psychiatry. 2025 Feb 4;16:1510255. doi: 10.3389/fpsyt.2025.1510255 (PMC11832497; doi:10.3389/fpsyt.2025.1510255)
Supplement: Supplementary file 1 [file DataSheet1.pdf]

Supplementary Materials

Contents

1. Figure S1. Accuracy of edge weights..2
2. Figure S2. Accuracy of edge weights… 3
3. Figure S3. Accuracy of edge weights… 4
4. Figure S4. Bootstrapped difference test for edge weights....................................5
5. Figure S5. Bootstrapped difference test for edge weights…..................................6
6. Figure S6. Bootstrapped difference test for edge weights…..................................7
7. Figure S7. Bootstrapped difference test for node strength....................................8
8. Figure S8. Bootstrapped difference test for node strength… 9
9. Figure S9. Bootstrapped difference test for node strength… 10
10. Figure S10. Edge, node strength and stability of bridge strength… 11
11. Figure S11. Edge, node strength and stability of bridge strength….......................12
12. Figure S12. Edge, node strength and stability of bridge strength… 13
13. Table A. Model comparison results...14
14. Table B. Summary of mean, standard deviation, skewness, and kurtosis of three groups…..........15
15. Table C. Edge weights of three groups 16
16. Table D. The centrality of nodes in the network for the three group 18

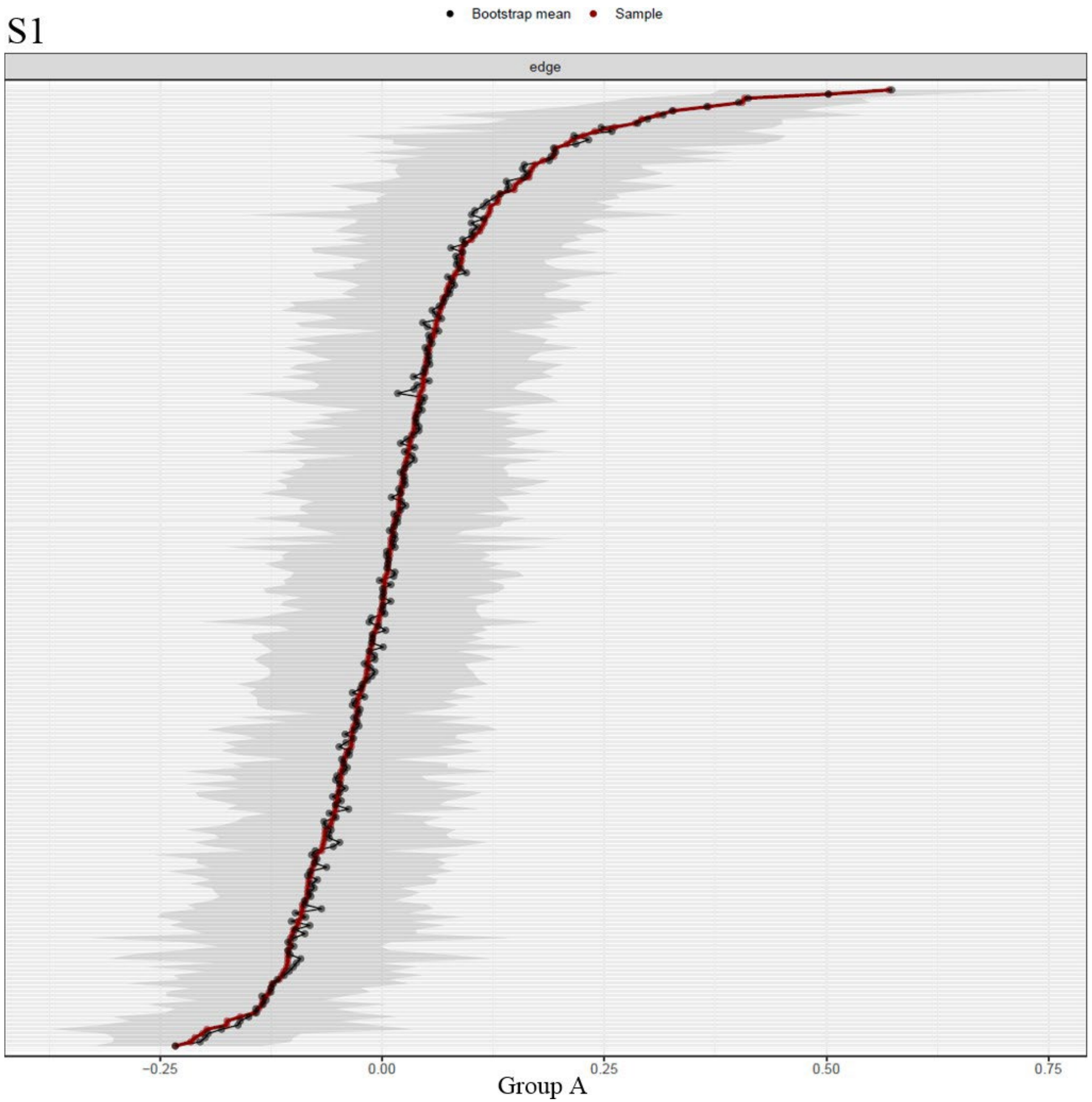

Figure S1. Accuracy of edge weights of Group A. *Note*: The red line depicts the sample edge weights and the gray bar depicts the bootstrapped confidence interval.

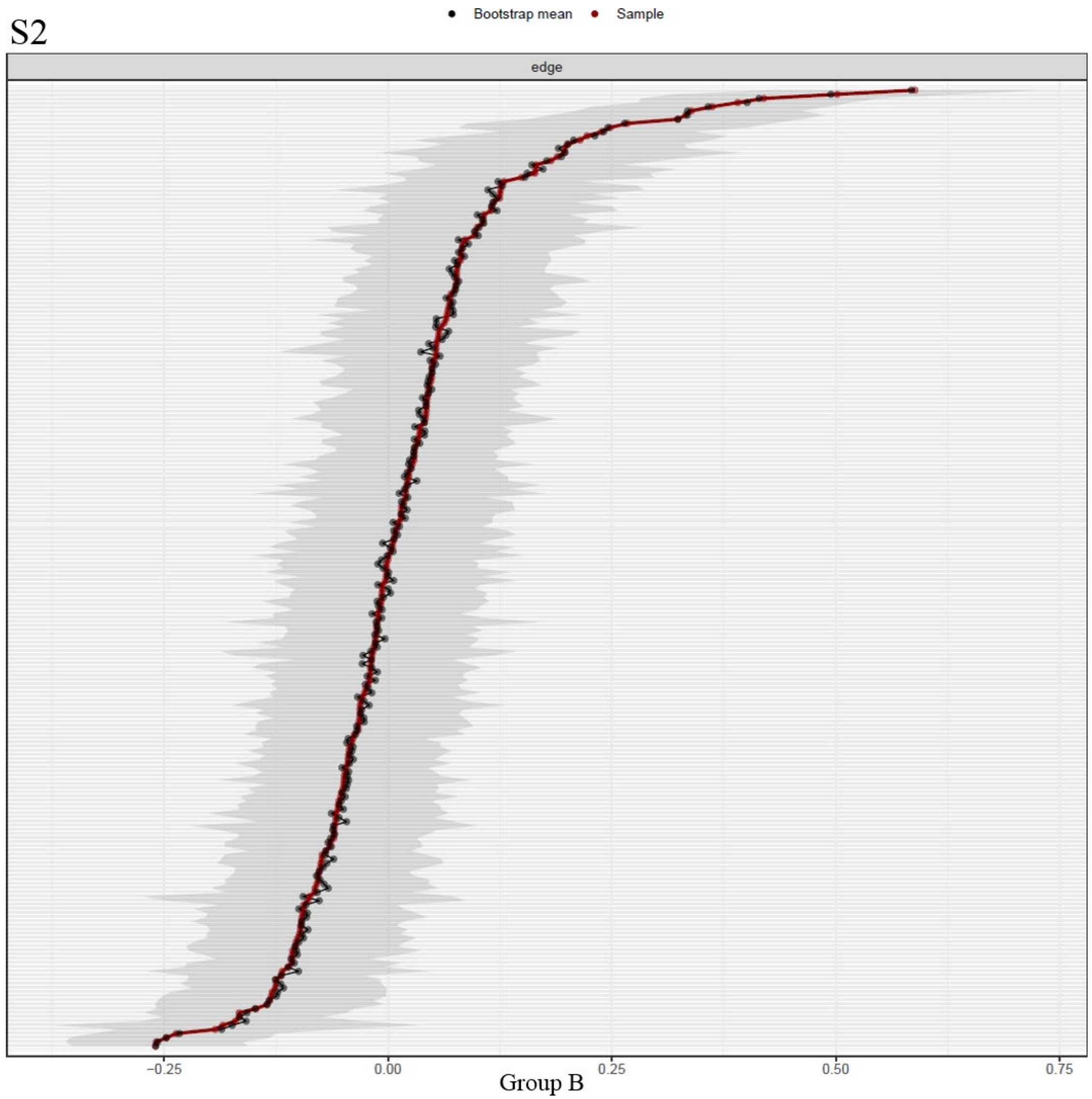

Figure S2. Accuracy of edge weights of Group B. *Note:* The red line depicts the sample edge weights and the gray bar depicts the bootstrapped confidence interval.

S3

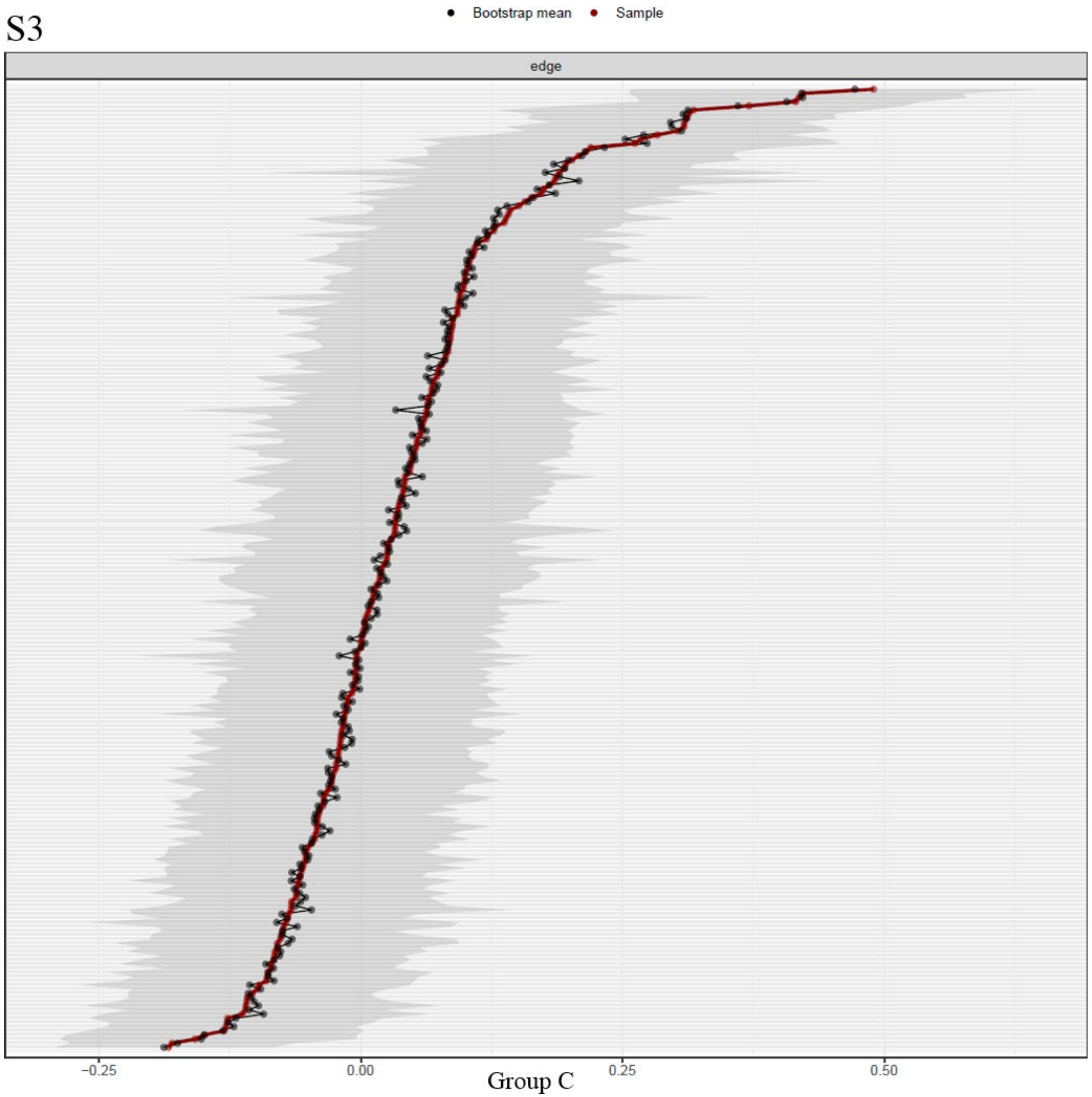

Figure S3. Accuracy of edge weights of Group C. *Note*: The red line depicts the sample edge weights and the gray bar depicts the bootstrapped confidence interval.

S4

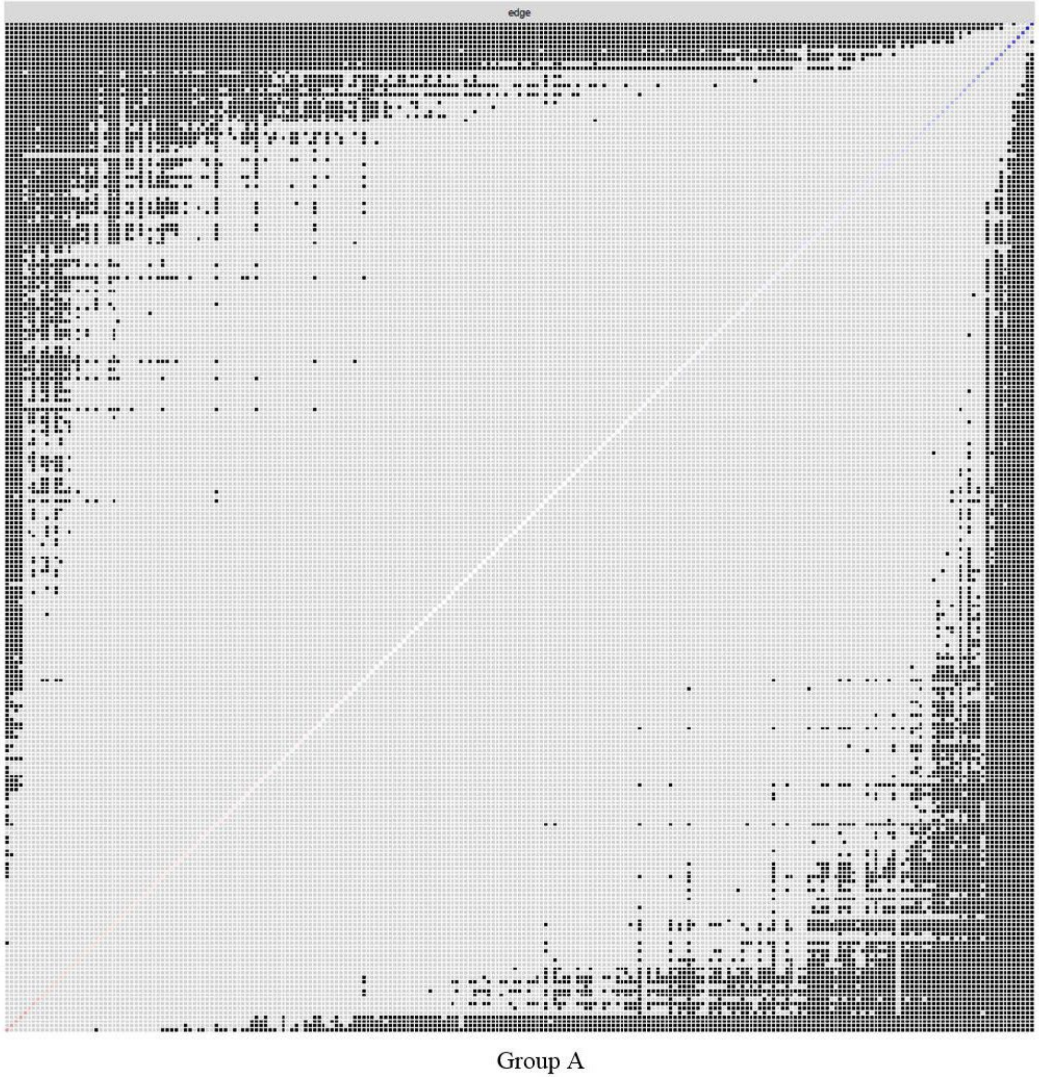

Figure S4. Bootstrapped difference test for edge weights of Group A. *Note*: Gray boxes indicate edge weights that do not differ significantly from one another, while black boxes indicate edge weights that do differ significantly. Blue and red boxes on the diagonal correspond to edge weights with positive and negative correlations, respectively.

S5

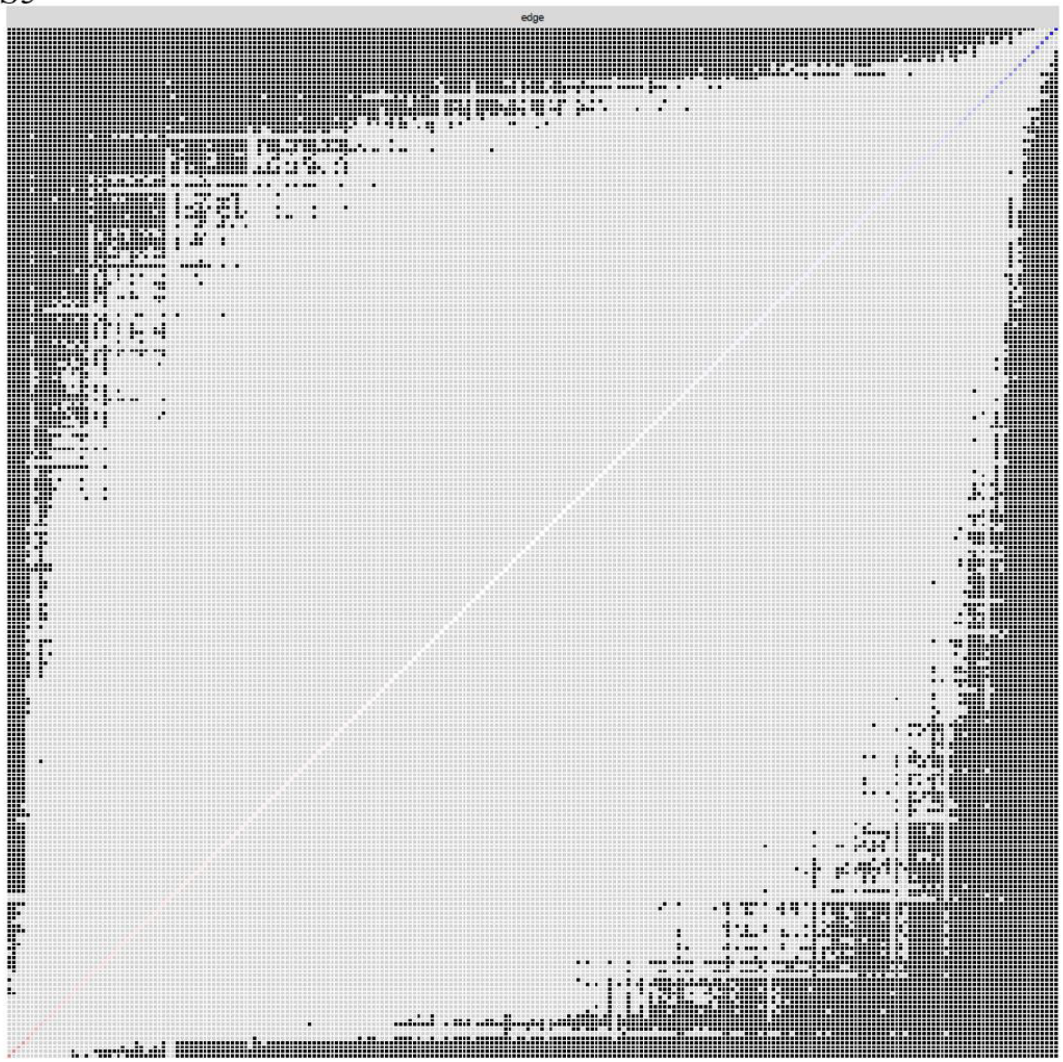

Group B

Figure S5. Bootstrapped difference test for edge weights of Group B. *Note*: Gray boxes indicate edge weights that do not differ significantly from one another, while black boxes indicate edge weights that do differ significantly. Blue and red boxes on the diagonal correspond to edge weights with positive and negative correlations, respectively.

S6

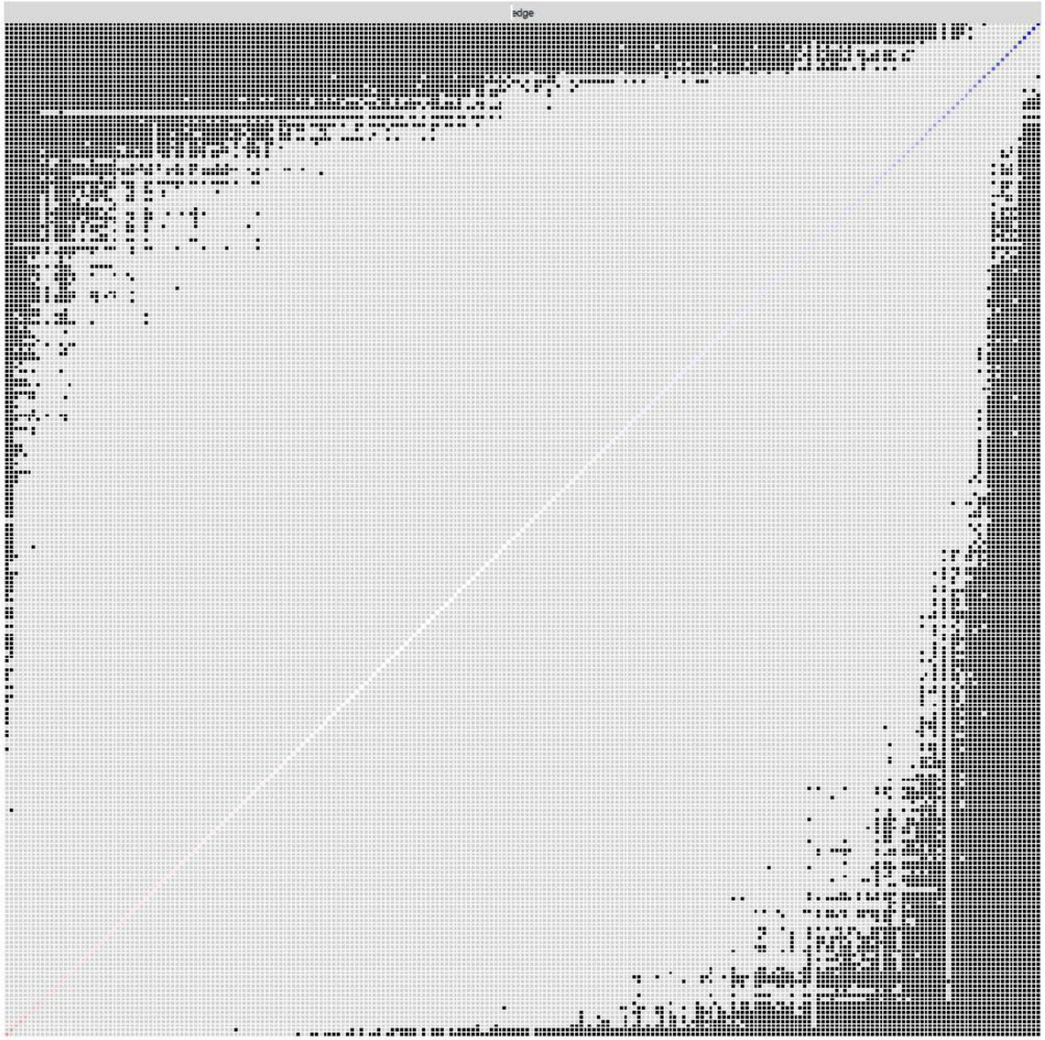

Group C

Figure S6. Bootstrapped difference test for edge weights of Group C. *Note*: Gray boxes indicate edge weights that do not differ significantly from one another, while black boxes indicate edge weights that do differ significantly. Blue and red boxes on the diagonal correspond to edge weights with positive and negative correlations, respectively.

S7

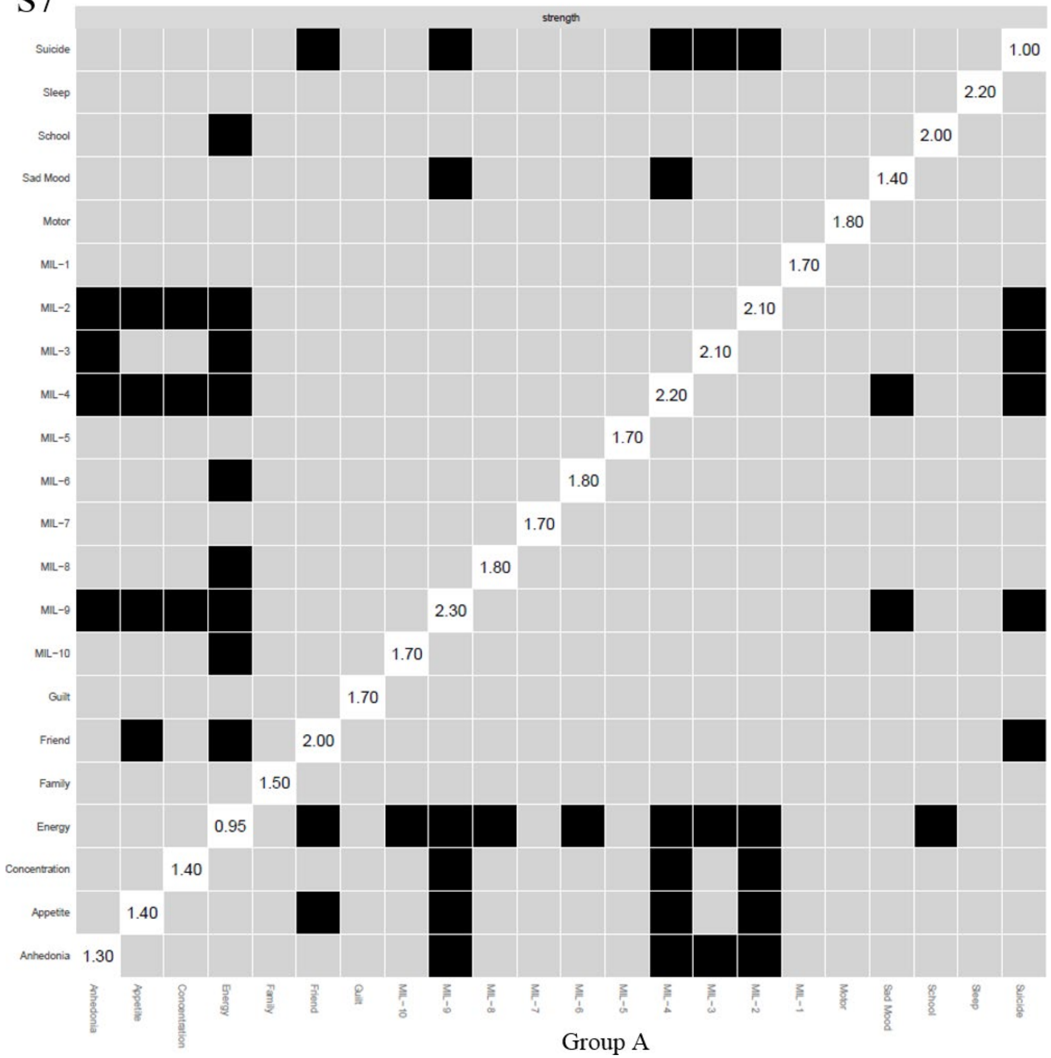

Figure S7. Bootstrapped difference test for node strength of Group A. *Note*: Gray boxes indicate expected influence of nodes that do not differ significantly from one another, while black boxes indicate expected influence that do differ significantly.

S8

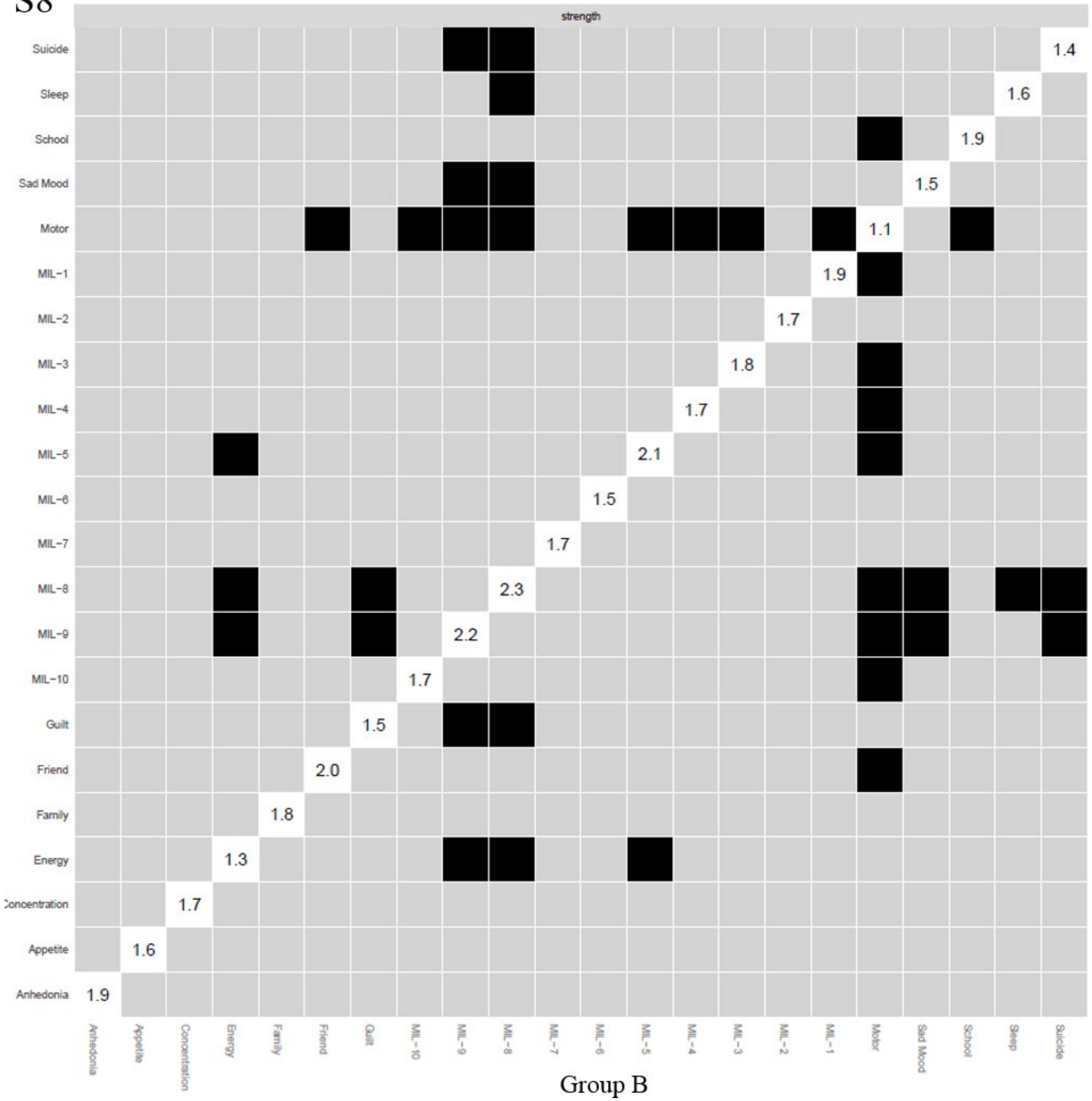

Figure S8. Bootstrapped difference test for node strength of Group B. *Note*: Gray boxes indicate expected influence of nodes that do not differ significantly from one another, while black boxes indicate expected influence that do differ significantly.

S9

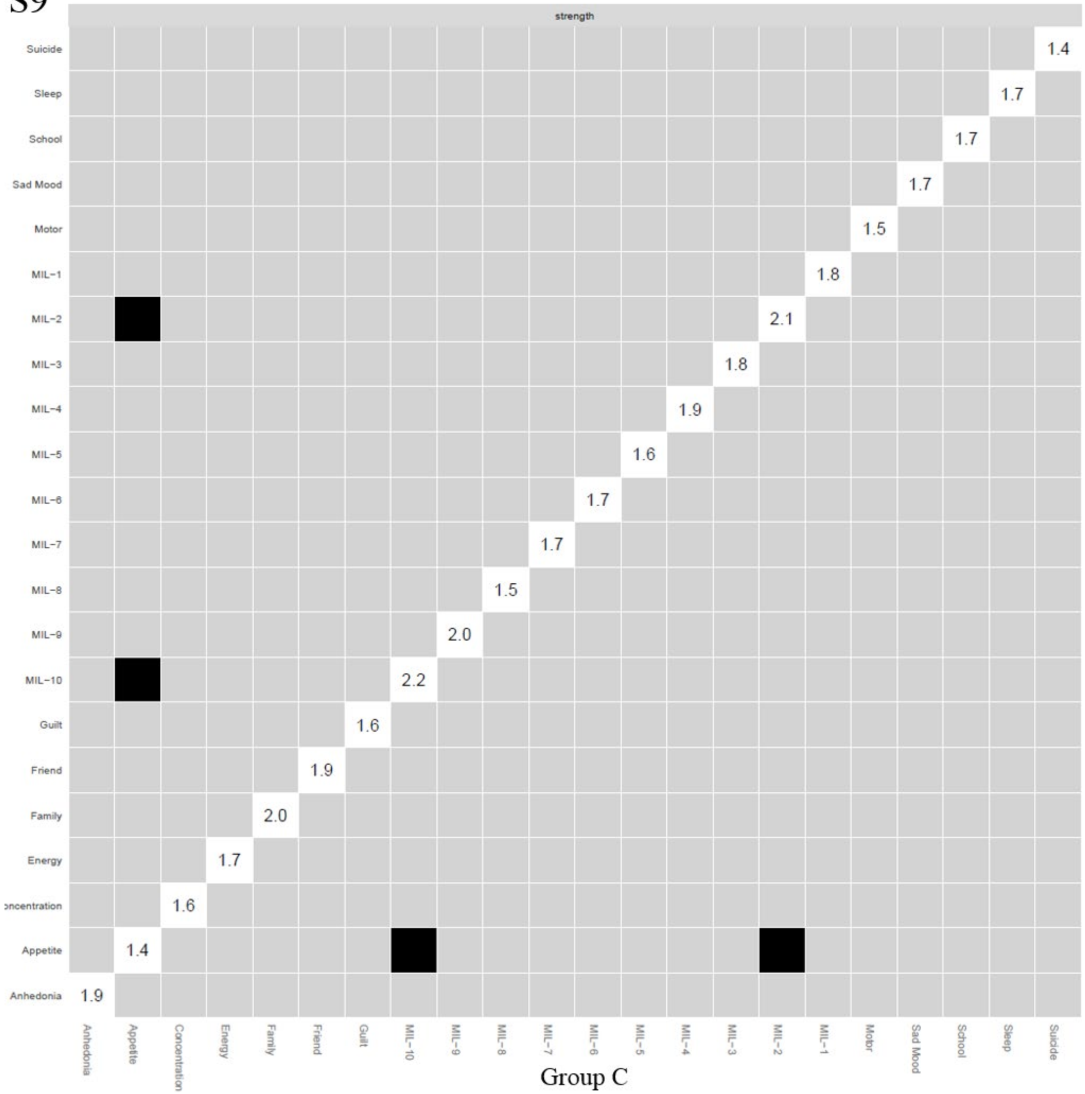

Figure S9. Bootstrapped difference test for node strength of Group C. *Note*: Gray boxes indicate expected influence of nodes that do not differ significantly from one another, while black boxes indicate expected influence that do differ significantly.

S10

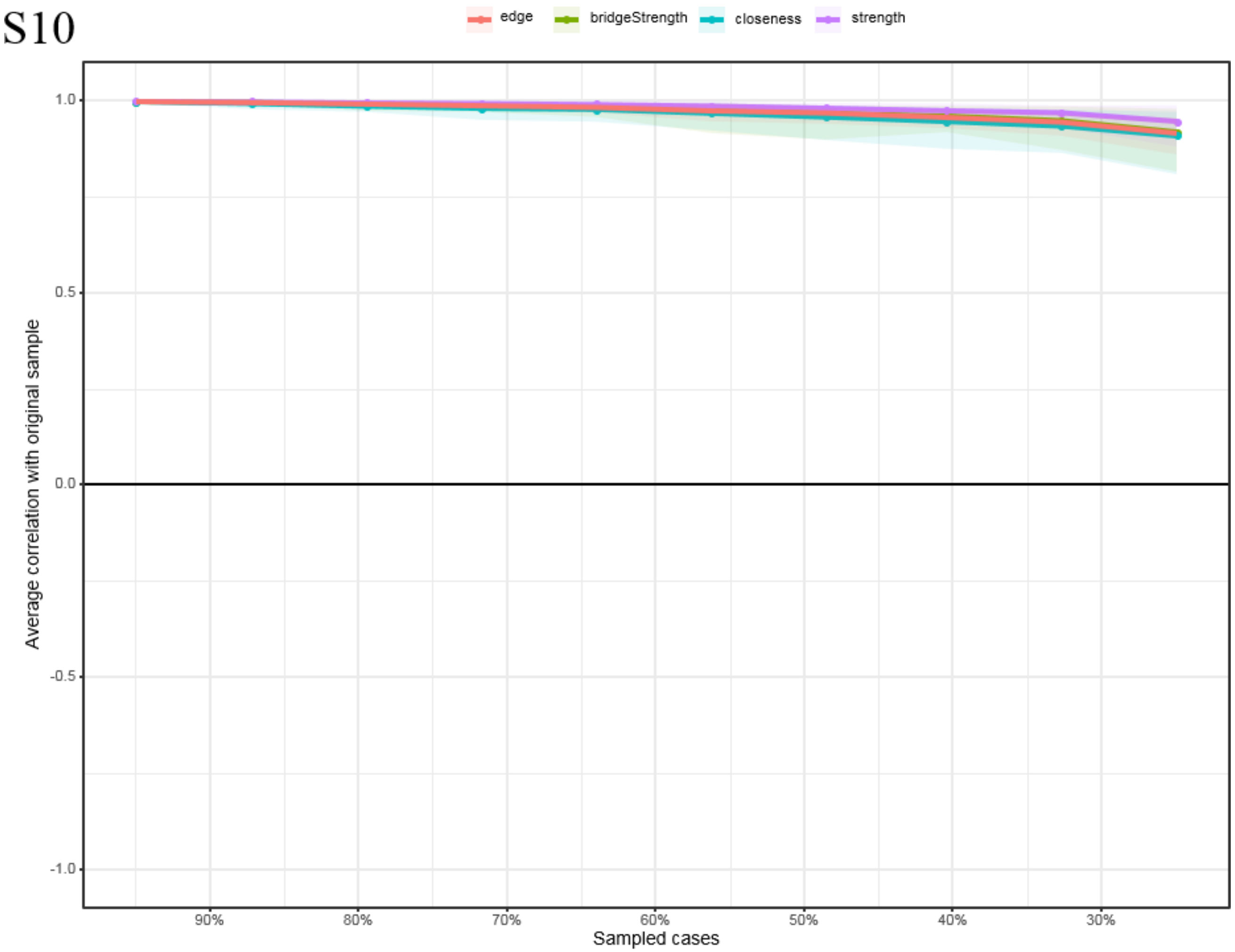

Group A

Figure S10. Edge, node strength, and stability of bridge strength of Group A. *Note*: The solid bars represent the average correlation of edge, node strength, and expected influence in the total and subsamples, with the shaded area describing the 2.5th to 97.5th quartile.

S11

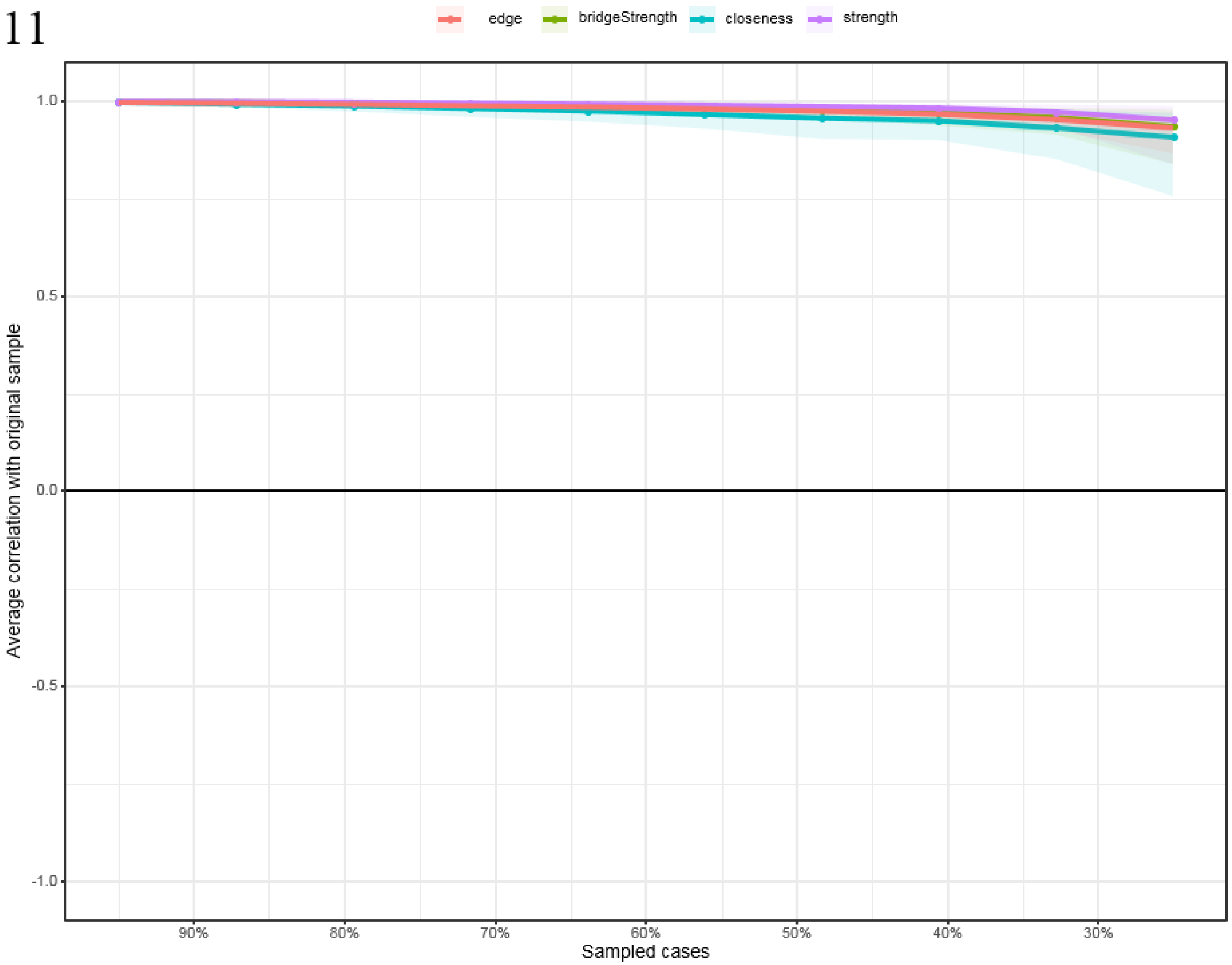

Group B

Figure S11. Edge, node strength, and stability of bridge strength of Group B. *Note*: The solid bars represent the average correlation of edge, node strength, and expected influence in the total and subsamples, with the shaded area describing the 2.5th to 97.5th quartile.

S12

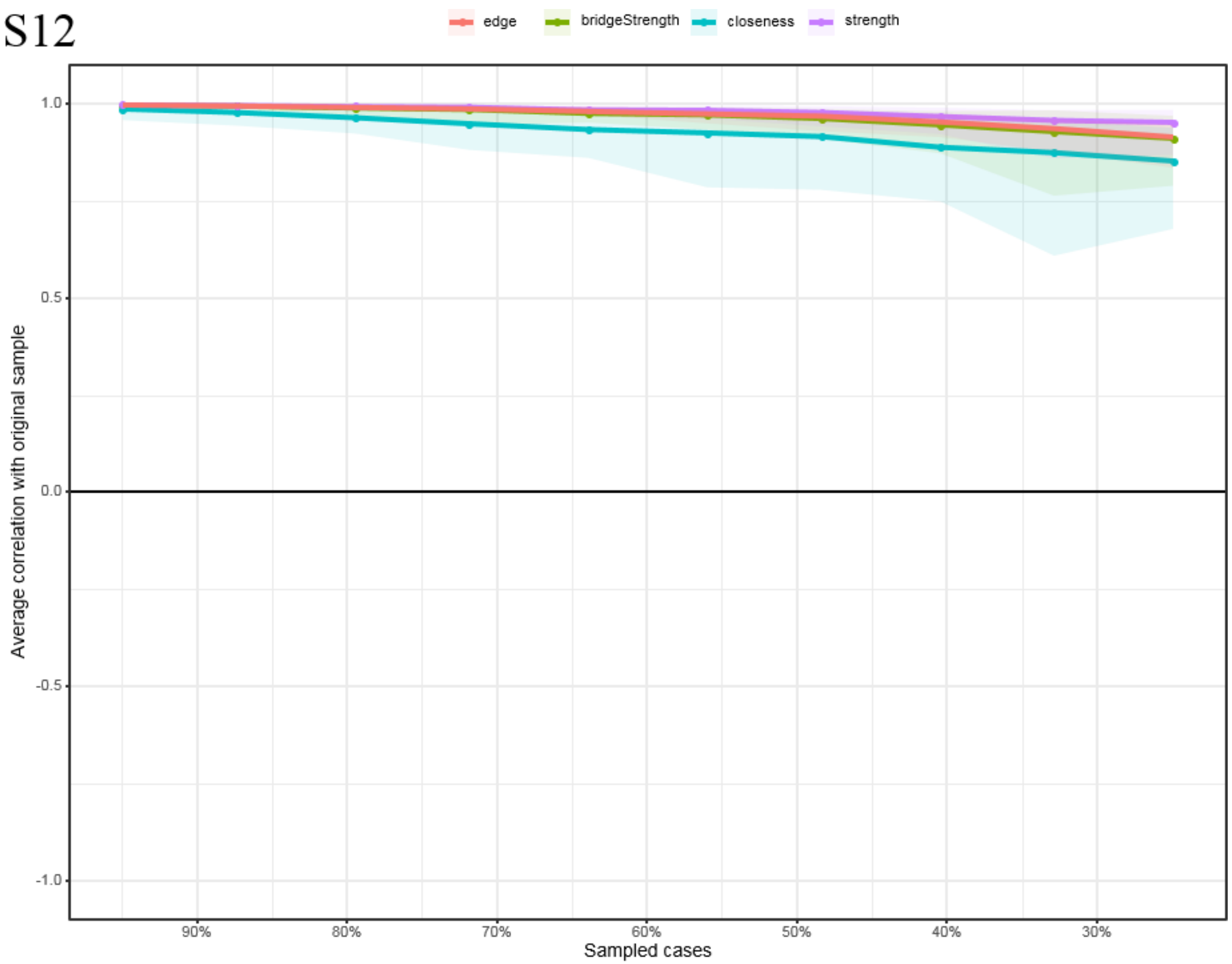

Group C

Figure S12. Edge, node strength, and stability of bridge strength of Group C. *Note*: The solid bars represent the average correlation of edge, node strength, and expected influence in the total and subsamples, with the shaded area describing the 2.5th to 97.5th quartile.

Table A | Model comparison results.

Models	χ^2	χ^2 / df	Df	CFI	TLI	RMSEA	SRMR	$\Delta\chi^2$	Δdf
Six-factor Model	1114.15	2.66	419	.967	.963	.035	.030	—	—
Five-factor Model	3267.07	7.71	424	.865	.852	.070	.089	2152.92	5
Four-factor Model (a)	4309.31	10.07	428	.815	.799	.081	.091	3195.16	9
Four-factor Model (b)	4146.99	9.69	428	.823	.808	.080	.092	3032.84	9
Three-factor Model	5108.48	11.85	431	.778	.760	.089	.094	3994.33	12
Two-factor Model	8429.17	19.47	433	.620	.592	.116	.134	7315.02	14
One-factor Model	11599.59	26.73	434	.469	.431	.137	.180	10485.14	15

Notes. "+" indicates that these factors are combined into one factor.
Six-factor Model: sense of significance + search for meaning + school + family+ friend + depression.
Five-factor Model: sense of significance, search for meaning + school + family + friend + depression.
Four-factor Model (a): sense of significance, search for meaning + school, family + friend + depression.
Four-factor Model (b): sense of significance, search for meaning + school + family, friend + depression.
Three-factor Model: sense of significance, search for meaning + school, family, friend + depression.
Two-factor Model: sense of significance, search for meaning, school, family, friend + depression.
One-factor Model: sense of significance, search for meaning, school, family, friend, depression.

Table B | Summary of mean, standard deviation, skewness, and kurtosis of each variable selected in the three depressive symptom networks.

Items	Short codes	Group A (N = 297)				Group B (N = 399)				Group C (N = 277)			
		M	*SD*	Skewness	Kurtosis	*M*	*SD*	Skewness	Kurtosis	*M*	*SD*	Skewness	Kurtosis
I understand my life's meaning.	MIL-1	4.60	1.29	-0.66	0.97	4.35	1.28	-0.29	0.60	4.20	1.47	-0.33	0.10
My life has a clear sense of purpose.	MIL-2	4.69	1.24	-0.45	0.66	4.38	1.23	-0.34	0.38	4.10	1.42	-0.23	-0.03
I have a good sense of what makes my life meaningful.	MIL-3	4.90	1.31	-0.62	0.76	4.47	1.26	-0.31	0.31	4.29	1.43	-0.32	0.01
I have discovered a satisfying life purpose.	MIL-4	4.43	1.34	-0.43	0.48	4.14	1.30	-0.32	0.28	3.88	1.47	-0.33	-0.32
My life has no clear purpose.	MIL-5	4.62	1.27	-0.48	0.84	4.36	1.25	-0.28	0.39	4.12	1.45	-0.23	-0.11
I am looking for something that makes my life feel meaningful.	MIL-6	4.94	1.32	-0.88	1.24	4.79	1.24	-0.50	0.55	4.88	1.39	-0.57	0.56
I am always looking to find my life's purpose.	MIL-7	4.67	1.37	-0.68	0.55	4.62	1.31	-0.42	0.10	4.64	1.41	-0.42	0.13
I am always searching for something that makes my life feel significant.	MIL-8	4.88	1.36	-0.64	0.62	4.84	1.33	-0.51	0.24	4.91	1.45	-0.60	0.30
I am seeking a purpose or mission for my life.	MIL-9	4.84	1.35	-0.80	0.89	4.80	1.28	-0.45	0.30	4.76	1.42	-0.57	0.29
I am searching for meaning in my life.	MIL-10	4.93	1.35	-0.75	0.75	4.83	1.30	-0.51	0.29	4.82	1.44	-0.57	0.21
There is a special person (eg., teacher, classmate) who is around when I am in need.	School-1	5.27	1.29	-1.20	1.71	4.74	1.45	-0.78	0.25	4.58	1.50	-0.56	-0.12
There is a special person (eg., teacher, classmate) with whom I can share joys and sorrows	School-2	5.38	1.29	-1.31	1.97	4.74	1.51	-0.76	-0.10	4.53	1.54	-0.54	-0.20
I have a special person (eg., teacher, classmate) who is a real source of comfort to me.	School-3	5.28	1.36	-1.20	1.31	4.60	1.53	-0.65	-0.11	4.44	1.60	-0.44	-0.47
There is a special person (eg., teacher, classmate) in my life who cares about my feelings.	School-4	5.36	1.30	-1.23	1.43	4.75	1.51	-0.74	-0.04	4.57	1.53	-0.50	-0.30
My family really tries to help me.	Family-5	5.59	1.24	-1.33	2.03	4.99	1.30	-0.63	0.16	4.79	1.45	-0.53	-0.16
I get the emotional help and support I need from my family.	Family-6	5.51	1.20	-1.09	1.44	5.12	1.36	-0.96	0.54	4.69	1.50	-0.46	-0.21
I can talk about my problems with my family.	Family-7	5.36	1.41	-1.09	0.83	4.88	1.37	-0.64	0.03	4.45	1.60	-0.44	-0.53
My family is willing to help me make decisions.	Family-8	5.52	1.35	-1.19	1.21	5.02	1.38	-0.72	0.00	4.64	1.52	-0.56	-0.21
My friends really try to help me.	Friend-9	5.51	1.16	-1.23	2.41	5.08	1.44	-0.67	-0.15	4.81	1.43	-0.58	-0.04
I can count on my friends when things go wrong.	Friend-10	5.63	1.11	-1.36	2.93	4.98	1.48	-0.74	-0.01	4.99	1.42	-0.74	0.18
I have friends with whom I can share my joys and sorrows.	Friend-11	5.42	1.25	-1.31	2.46	4.74	1.61	-0.57	-0.40	4.73	1.48	-0.56	-0.08
I can talk about my problems with my friends.	Friend-12	5.53	1.20	-1.40	2.75	4.95	1.57	-0.68	-0.25	4.82	1.57	-0.58	-0.22
Little interest or pleasure in doing things.	Anhedonia	0.51	0.55	0.41	-0.98	1.02	0.52	0.91	4.14	1.75	0.76	0.16	-0.73
Feeling down, depressed, or hopeless.	Sad Mood	0.34	0.52	1.10	0.10	0.85	0.50	-0.30	0.59	1.60	0.78	0.18	-0.51
Trouble falling or staying asleep, or sleeping too much.	Sleep	0.33	0.59	1.90	3.96	0.83	0.69	0.56	0.34	1.65	0.92	-0.13	-0.81
Feeling tired or having little energy.	Energy	0.53	0.55	0.39	-0.93	1.01	0.53	0.51	2.50	1.96	0.72	0.00	-0.92
Poor appetite or overeating.	Appetite	0.20	0.44	2.34	6.59	0.75	0.63	0.67	1.38	1.60	0.87	-0.08	-0.65
Feeling bad about yourself- or that you are a failure or have let yourself or your family down.	Guilt	0.14	0.40	3.26	12.74	0.75	0.63	0.48	0.57	1.57	0.85	0.00	-0.61
Trouble concentrating on things, such as reading the newspaper or watching television.	Concentration	0.33	0.55	1.64	3.16	1.03	0.73	0.85	1.23	1.77	0.82	-0.23	-0.45
Moving or speaking so slowly that other people could have noticed? Or the opposite being so fidgety or restless that you have been moving around a lot more than usual.	Motor	0.08	0.29	3.52	12.25	0.60	0.55	0.23	-0.33	1.49	0.89	0.00	-0.72
Thoughts that you would be better off dead or of hurting yourself in some way.	Suicide	0.01	0.08	12.12	145.97	0.27	0.48	1.67	2.78	0.90	0.96	0.65	-0.77

Table C | Edge weights of three groups.

Group A		MIL-1	MIL-2	MIL-3	MIL-4	MIL-5	MIL-6	MIL-7	MIL-8	MIL-9	MIL-10	Anhedonia	Sad Mood	Sleep	Energy	Appetite	Guilt	Concentration	Motor	Suicide	School	Family	Friend
	**MIL-1**	.00																					
	**MIL-2**	.41	.00																				
	**MIL-3**	.08	.29	.00																			
	**MIL-4**	.26	.07	.29	.00																		
	**MIL-5**	-.06	.33	.13	.37	.00																	
	**MIL-6**	-.05	.06	.17	-.07	.03	.00																
	**MIL-7**	.10	-.07	-.05	.01	-.05	.21	.00															
	**MIL-8**	.00	.07	-.10	-.11	.17	.18	.23	.00														
	**MIL-9**	-.10	.05	.05	.01	-.04	.12	.31	.21	.00													
	**MIL-10**	.07	-.06	.02	.03	.04	.19	.00	.15	.57	.00												
	**Anhedonia**	-.02	-.04	.06	-.09	.02	.05	.00	.03	-.05	.05	.00											
	**Sad Mood**	-.03	.13	-.12	-.11	.05	-.03	-.07	.00	.00	.01	.13	.00										
	**Sleep**	-.12	.08	.12	-.08	-.01	-.09	.04	.09	-.20	.17	-.20	-.08	.00									
	**Energy**	.07	-.02	.05	-.02	.02	-.05	.09	.04	-.10	.00	.04	.01	-.10	.00								
	**Appetite**	.00	-.08	.02	.03	-.03	.10	-.08	.09	-.09	.03	-.14	-.14	-.11	.00	.00							
	**Guilt**	.05	-.08	.08	-.08	.01	.11	-.05	-.11	-.06	.15	-.17	.06	-.13	-.08	-.11	.00						
	**Concentration**	-.08	.04	.09	-.11	-.03	.02	-.01	.02	-.11	-.01	.00	-.23	-.22	-.02	-.05	-.02	.00					
	**Motor**	.01	-.09	.15	.01	-.01	-.10	.07	.09	-.06	.04	-.03	.03	-.13	-.13	-.10	.01	.17	.00				
	**Suicide**	.03	.01	.00	-.02	.04	-.05	.06	.01	-.05	.04	-.07	-.04	-.03	-.05	.07	.04	-.03	.24	.00			
	**School**	.11	.05	.12	-.21	.06	.00	-.08	-.02	.08	.02	-.04	.05	.09	-.01	-.03	-.17	.05	-.09	.01	.00		
	**Family**	-.01	.09	.06	.03	-.14	-.03	.04	.05	-.02	.03	.00	-.09	-.05	-.03	-.01	.02	-.05	-.16	.01	.19	.00	
	**Friend**	-.08	-.03	-.03	.20	-.02	-.01	.11	.01	-.06	.02	-.04	.02	-.03	.01	.02	.06	.05	.12	-.13	.50	.41	.00
**Group B**	**MIL-1**	.00																					
	**MIL-2**	.50	.00																				
	**MIL-3**	.16	.10	.00																			
	**MIL-4**	.06	.07	.27	.00																		
	**MIL-5**	.13	.25	.22	.33	.00																	
	**MIL-6**	.05	-.03	.20	.20	-.09	.00																
	**MIL-7**	.03	-.01	-.09	.02	.05	.21	.00															
	**MIL-8**	-.12	.02	-.02	-.11	.12	.19	.32	.00														
	**MIL-9**	.08	.12	.09	.00	-.18	.02	.18	.36	.00													
	**MIL-10**	-.07	-.05	.00	-.01	.10	.15	.02	.13	.59	.00												
	**Anhedonia**	.08	-.01	-.13	-.06	-.02	.01	-.13	.12	-.02	.02	.00											
	**Sad Mood**	-.10	.07	.00	.03	.01	-.07	.05	-.09	.07	-.01	.20	.00										
	**Sleep**	.05	.08	.08	-.04	-.03	-.01	.13	-.06	-.01	-.05	-.10	-.03	.00									
	**Energy**	.04	.02	.08	-.11	-.08	-.01	.05	-.03	-.06	.05	.11	-.05	.02	.00								
	**Appetite**	.05	.07	-.01	.01	-.10	-.03	.03	-.08	-.08	.08	-.26	-.10	-.13	-.10	.00							
	**Guilt**	-.03	-.02	.08	.01	-.11	-.01	.03	.07	-.04	-.01	-.17	.05	-.24	-.13	-.11	.00						
	**Concentration**	.02	.00	.04	-.01	-.04	-.06	.17	-.10	-.02	.03	-.10	-.25	-.26	-.08	-.19	.00	.00					

	**Motor**	-.05	.04	-.03	.12	-.05	-.01	-.04	.04	-.02	.01	-.07	-.03	-.12	-.01	-.10	.07	.01	.00				
	**Suicide**	-.13	.11	-.08	-.01	.06	.03	.05	-.07	.01	-.04	-.05	-.02	-.04	-.17	.02	.04	-.11	.24	.00			
	**School**	.08	.04	-.04	.04	.00	-.05	-.06	.07	-.08	.16	-.10	.08	-.05	.05	.00	.04	-.08	-.03	.03	.00		
	**Family**	-.02	.06	-.03	.07	-.06	.05	.04	.08	-.02	-.09	-.04	.04	-.06	-.06	-.06	-.17	-.02	.02	-.08	.34	.00	
	**Friend**	-.05	.06	.08	-.15	.10	.01	-.02	-.14	.11	.00	.11	-.12	-.01	.01	.06	.03	.05	.00	-.06	.42	.39	.00
**Group C**	**MIL-1**	.00																					
	**MIL-2**	.37	.00																				
	**MIL-3**	.31	.17	.00																			
	**MIL-4**	-.04	.30	.20	.00																		
	**MIL-5**	.07	.28	.10	.42	.00																	
	**MIL-6**	.05	.06	.09	-.11	.04	.00																
	**MIL-7**	.03	-.03	.06	-.09	.16	.02	.00															
	**MIL-8**	.09	-.02	.03	-.07	-.02	.26	.27	.00														
	**MIL-9**	.00	.12	-.07	-.04	.01	.22	.04	.19	.00													
	**MIL-10**	-.11	-.08	.06	.11	.01	.14	.32	.19	.49	.00												
	**Anhedonia**	.13	-.11	-.04	.06	-.03	.14	-.07	.05	-.05	-.05	.00											
	**Sad Mood**	.03	-.01	.03	.00	-.08	-.09	-.13	.04	.15	.03	.31	.00										
	**Sleep**	-.06	.09	.05	-.03	.01	.04	-.02	-.04	-.18	.20	.06	.11	.00									
	**Energy**	-.03	.01	-.02	-.06	.01	.06	.10	.02	.04	-.15	.16	.09	.31	.00								
	**Appetite**	-.04	-.06	-.02	.05	.00	.14	.05	-.01	-.11	.02	.05	.06	-.04	.08	.00							
	**Guilt**	-.13	.09	.09	-.06	-.09	-.02	.07	.06	.01	.00	-.05	.11	.08	-.02	.14	.00						
	**Concentration**	.01	-.04	.05	-.06	-.02	-.08	-.07	.00	.09	.05	.09	-.05	-.09	.18	-.04	.02	.00					
	**Motor**	.07	-.07	-.07	.10	-.01	-.02	-.01	-.02	.03	-.01	.03	.06	.02	.04	.07	.17	.31	.00				
	**Suicide**	.02	.00	-.10	.04	.10	-.06	.10	-.09	.00	-.04	.04	.07	.07	-.08	.04	.19	.10	.19	.00			
	**School**	-.01	.10	.08	-.03	-.02	-.04	-.06	-.03	.09	.02	.07	-.08	-.05	-.08	.22	.03	.07	.00	-.02	.00		
	**Family**	-.13	-.02	.09	.00	.05	.03	.00	.00	-.07	.12	.13	-.10	-.11	.08	-.08	-.01	-.16	11	-.10	.21	.00	
	**Friend**	.08	-.07	-.04	.03	.03	.00	-.01	.00	-.02	.00	-.18	.09	.09	.10	-.05	-.13	.08	-.06	.00	.42	.42	.00

Table D | The centrality of nodes in the network for the three group.

Nodes	*Strength*			*Bridge Strength*		
	Group A (*N* = 297)	Group B (*N* = 399)	Group C (*N* = 277)	Group A (*N* = 297)	Group B (*N* = 399)	Group C (*N* = 277)
MIL-1	1.744	1.898	1.816	0.93	1.049	1.026
MIL-2	2.136	1.719	2.086	1.049	0.804	0.956
MIL-3	2.071	1.821	1.806	1.281	1.069	1.018
MIL-4	2.202	1.711	1.9	1.213	0.987	0.931
MIL-5	1.653	2.134	1.57	0.764	1.206	0.692
MIL-6	1.754	1.491	1.726	1.046	0.917	1.081
MIL-7	1.715	1.723	1.711	0.959	0.989	1.058
MIL-8	1.769	2.338	1.493	1.004	1.338	0.588
MIL-9	2.322	2.152	2.026	1.114	0.999	1.084
MIL-10	1.708	1.674	2.208	0.79	0.796	1.076
Anhedonia	1.292	1.913	1.897	0.508	0.853	1.099
Sad Mood	1.434	1.484	1.705	0.711	0.75	0.839
Sleep	2.171	1.593	1.742	1.17	0.643	0.956
Energy	0.951	1.297	1.733	0.522	0.628	0.765
Appetite	1.355	1.638	1.376	0.623	0.633	0.852
Guilt	1.663	1.455	1.588	1.043	0.662	0.798
Concentration	1.404	1.658	1.634	0.663	0.654	0.762
Motor	1.829	1.139	1.466	0.994	0.47	0.581
Suicide	1.026	1.444	1.45	0.455	0.754	0.667
School	1.979	1.871	1.726	1.979	1.871	1.726
Family	1.523	1.799	2.008	1.523	1.799	2.008
Friend	1.971	1.973	1.924	1.971	1.973	1.924
